# A designer protein binder of KRAS enables exogenous protein degradation

**DOI:** 10.1002/pro.70850

**Published:** 2026-11-09

**Authors:** Aniekan Okon, Keith M. Cheah, Oscar J. Molina, Gabriel Saenz, K. Dane Wittrup, Ronald T. Raines

**Affiliations:** ^1^ Department of Chemistry Massachusetts Institute of Technology Cambridge Massachusetts USA; ^2^ Department of Chemical Engineering Massachusetts Institute of Technology Cambridge Massachusetts USA; ^3^ Department of Biology Massachusetts Institute of Technology Cambridge Massachusetts USA; ^4^ Koch Institute for Integrative Cancer Research at MIT Cambridge Massachusetts USA; ^5^ Department of Biological Engineering Massachusetts Institute of Technology Cambridge Massachusetts USA

**Keywords:** α‐aryl‐α‐diazoamide, bioconjugation, biodegrader, cellular delivery, PROTAC, Sso7d scaffold, VHL E3 ubiquitin ligase

## Abstract

Targeted protein degradation has emerged as a powerful strategy for modulating intracellular proteins, particularly those lacking traditional small‐molecule binding pockets. While protein‐based degraders expand this approach to undruggable targets, they typically require intracellular expression in target cells, limiting their utility as exogenous research tools. Here, we engineer the Sso7d‐derived KRAS^G12D^ binder R11.1.6 for use as a chemically conjugatable, exogenously deliverable degrader scaffold. We show that a C‐terminal arginine‐to‐cysteine variant can be produced in good yield and purity and conjugated to a von Hippel–Lindau (VHL) E3 ligase ligand, albeit with an approximately 10‐fold reduction in KRAS^G12D^ affinity relative to parent R11. To reduce the number of lysines available for potential ubiquitination, we generate two lysine‐reduced variants—a fully lysine‐free variant (R11^ΔLys^) and a “lysine‐lite” variant retaining only the three lysines implicated in KRAS engagement (R11^Lite^)—and demonstrate that both retain nanomolar affinity for wild‐type KRAS and KRAS^G12D^. Esterification of R11–VHL with an α‐aryl‐α‐diazoamide reagent enables cellular treatment with the chimeric conjugate and leads to VHL‐ligand‐dependent reduction in pan‐RAS levels in HPAF‐II cells, as shown by competition with excess free VHL ligand. Our results establish R11 variants as tractable protein‐engineering scaffolds for developing chemically conjugated biodegraders and provide design principles for engineering lysine‐reduced binders based on the Sso7d scaffold.

## 1 INTRODUCTION

Targeted protein degradation has emerged as a powerful strategy for controlling intracellular protein levels by harnessing the ubiquitin–proteasome system. Heterobifunctional molecules such as proteolysis‐targeting chimeras (PROTACs) recruit an E3 ubiquitin ligase to a protein of interest (POI), inducing polyubiquitination and proteasomal degradation in a catalytic manner (Sakamoto et al., 2001; Schneekloth Jr. et al., 2004; Bondeson et al., 2015; Lu et al., 2015; Winter et al., 2015) (Figure 1a). While small‐molecule PROTACs have proven effective for ligandable targets, their development against undruggable proteins, including transcription factors, scaffolding proteins, and proteins lacking deep small‐molecule binding pockets, remains challenging (Li et al., 2023; Liu & Ciulli, 2023).

**FIGURE 1:**
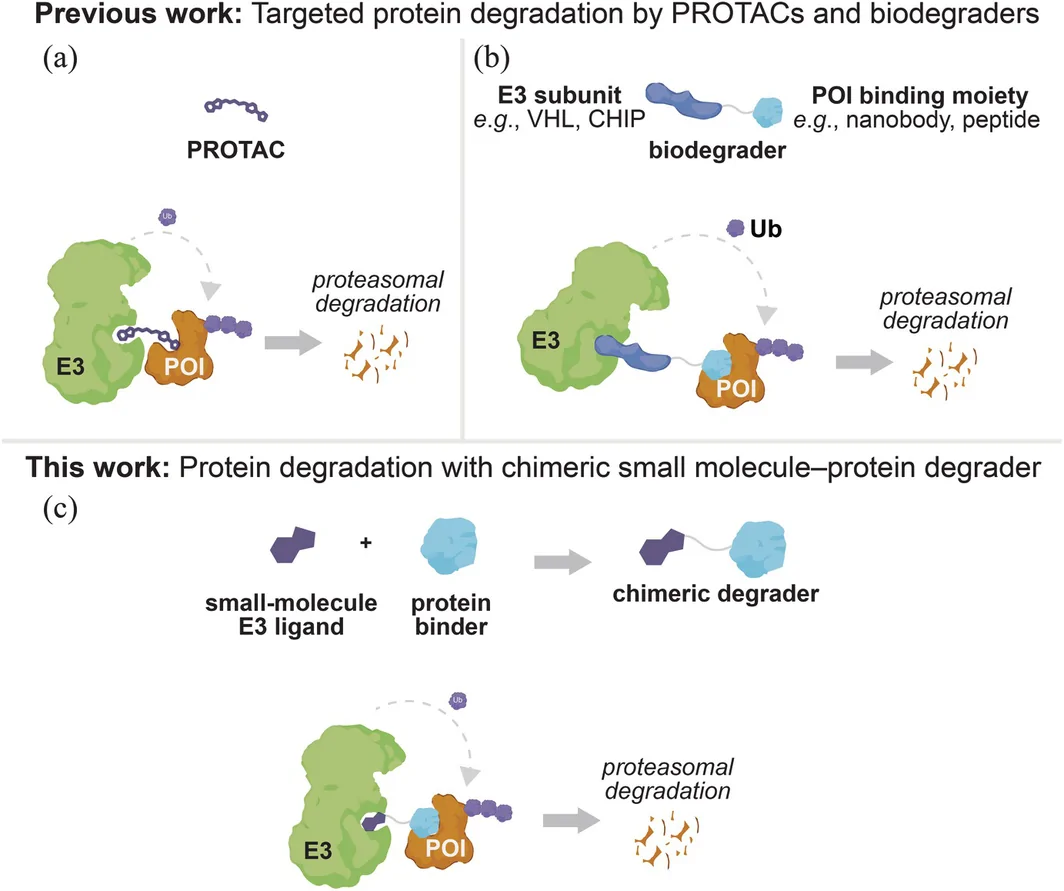
A chimeric degrader complements small‐molecule PROTACs and intracellularly expressed biodegraders for targeted protein degradation. (a,b) Conventional formats: Small‐molecule PROTACs that recruit ligandable proteins to an E3 ubiquitin ligase (a), and intracellularly expressed bioPROTACs that fuse a protein binder of the POI to an E3 ligase recruiter (b). (c) The chimeric degrader format introduced in this work, comprising a designer protein binder of a POI conjugated to a small‐molecule E3 ubiquitin ligase ligand. POI, protein of interest.

Protein‐based degraders, comprising a fusion of a POI‐binding protein and an E3 ligase recruiter, address this gap by leveraging the diversity of protein binders generated through directed evolution and rational design (Caussinus et al., 2011; Lim et al., 2020; Ludwicki et al., 2019; Portnoff et al., 2014; Simpson et al., 2020; VanDyke et al., 2022) (Figure 1b). Nonetheless, current protein‐based degraders are predominantly delivered by intracellular expression, limiting their utility as exogenous research tools and complicating translation. Recent efforts have begun to address this limitation through cell‐penetrating peptide fusions and engineered cell‐permeant scaffolds (Schmit et al., 2019; Shen et al., 2022). Recently, Plückthun and coworkers showed that microinjecting preformed bioPROTAC–target complexes enables rigorous single‐cell measurement of degradation rates across a wide range of E3 recruitment domains, peptide degrons, and small‐molecule warheads (Vukovic et al., 2026), providing valuable benchmarks for the field. Still, a general, modular strategy for chemically equipping a protein binder with an E3 ligase recruiter and then delivering that conjugate to cells in a scalable manner remains underdeveloped.

KRAS proteins, particularly oncogenic variants, are paradigmatic hard‐to‐drug targets (Papke & Der, 2017; Stephen et al., 2014). While covalent small‐molecule inhibitors have been developed for specific proteoforms, such as G12C (Fell et al., 2020; Janes et al., 2018; Lanman et al., 2020; Lim et al., 2014; Ostrem & Shokat, 2022; Zeng et al., 2017), G12D (Zheng et al., 2024), and G12S (Zhang et al., 2022), reversible inhibition of most KRAS variants relies on protein‐based binders (Guillard et al., 2017; Kauke et al., 2017; Spencer‐Smith et al., 2017). Among these, R11.1.6 (R11) is a thermostable 7‐kDa binder evolved from the Sso7d scaffold that engages KRAS^G12D^ with 4 nM affinity, representing a 10‐fold preference over KRAS^WT^ (Kauke et al., 2017). R11 has several attractive features as an engineering scaffold for biodegrader development: it is small, thermostable, cysteine‐free (enabling site‐specific bioconjugation via introduced cysteines), and produced in *Escherichia coli* in good yield.

Adapting R11 for use as an exogenously deliverable chimeric degrader requires solving three protein‐engineering problems: site‐specific introduction of a bioconjugation handle without disrupting the KRAS‐binding interface, cellular delivery of the resulting protein–small‐molecule conjugate, and mitigation of self‐degradation arising from ubiquitination of surface lysine residues on the binder. We note that VHL‐based biodegraders have been particularly effective for RAS family proteins in prior intracellularly expressed formats (Bond et al., 2020; Lim et al., 2021; Röth et al., 2020; Teng et al., 2021), motivating our choice of the VHL ligand as the E3 recruiter in this work.

Here, we explore whether a designer protein binder can be chemically coupled to a small‐molecule E3 ligase ligand and delivered exogenously to reduce intracellular target abundance (Figure 1c). Here, we report (1) the design and characterization of N‐ and C‐terminal cysteine variants of R11 and the trade‐offs each imposes; (2) the engineering of two lysine‐reduced R11 variants designed to limit lysine‐dependent ubiquitination with all or most lysines replaced with arginines, demonstrating that the Sso7d‐based KRAS‐binding interface tolerates extensive K → R substitution; (3) cellular delivery of an R11–VHL conjugate via protein esterification (Mix et al., 2017; Okon et al., 2025; Ressler et al., 2019) and (4) demonstration that the resulting chimeric degrader induces VHL‐dependent loss of pan‐RAS in HPAF‐II cells. We discuss the design principles, limitations, and future directions emerging from this work as a foundation for engineering exogenously deliverable protein‐based degraders.

## 2 RESULTS

### 2.1 Design and expression of R11 variants for chemical conjugation

R11 engages KRAS^G12D^ primarily through residues in its β‐sheet face near the C‐terminus, with switch II of KRAS as the principal interaction surface (Figure 2a) (Kauke et al., 2017). To preserve this interface while enabling site‐specific bioconjugation, we initially designed an N‐terminal cysteine variant (Cys‐R11), reasoning that modification distal to the binding interface would minimally perturb affinity. Recombinant expression of Cys‐R11 in *E. coli*, however, yielded the protein at low levels (0.5 mg L^−1^) and with modest purity (80%), rendering it impractical for downstream applications.

**FIGURE 2:**
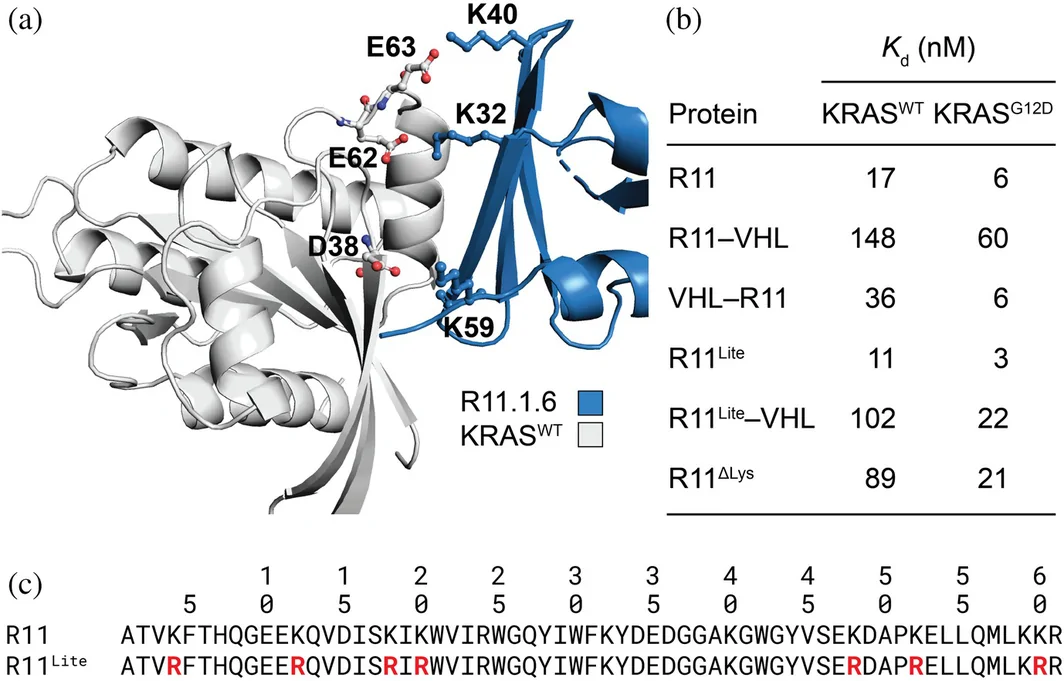
The Sso7d‐derived KRAS‐binding interface tolerates extensive lysine‐to‐arginine substitution. (a) Co‐crystal structure of R11 (cyan) bound to KRAS^G12D^ (gray); the three lysine residues (Lys32, Lys40, and Lys59) that contact KRAS are shown as sticks (PDB entry 5UFE). (b) Equilibrium dissociation constants (*K*_d_) for R11, R11^Lite^, R11^ΔLys^, and the indicated VHL conjugates against KRAS^WT^ and KRAS^G12D^, measured by bio‐layer interferometry. (c) Amino acid sequences of R11 and R11^Lite^, with lysine‐to‐arginine substitutions highlighted in red. R11^Lite^ retains the three interface lysines.

We therefore tested a C‐terminal cysteine variant (R11‐Cys), in which the terminal Arg was replaced with Cys, recognizing that modification at this site could perturb the binding interface. R11‐Cys was produced in 7‐fold higher yield (3.5 mg L^−1^) and >95% purity, even when purified as the 2‐nitro‐5‐thiobenzoate (NTB)‐protected species. Bio‐layer interferometry (BLI) measurements showed that the R11‐Cys–VHL conjugate bound KRAS^G12D^ ~ 10‐fold more weakly than parent R11 (Figure 2b), consistent with perturbation near the binding interface by the terminal R61C substitution, the appended VHL ligand, or both. Despite this affinity loss, the dramatic improvement in expression yield led us to adopt R11‐Cys as the primary scaffold for subsequent work.

### 2.2 Engineering ubiquitination‐resistant R11 variants

A practical limitation of using a small protein binder as a degrader scaffold is that the binder itself contains surface lysines that may be ubiquitinated and targeted for proteasomal degradation alongside the POI. R11 contains 10 lysine residues, three of which (Lys32, Lys40, and Lys59) form direct contacts with KRAS^G12D^ in a co‐crystal structure (PDB entry 5UFE) (Kauke et al., 2017). To generate variants with fewer lysine ubiquitination sites, we replaced surface lysines with arginines, a substitution that preserves the formal positive charge while eliminating the primary amine substrate of E3 ubiquitin ligases (Molina et al., 2026).

We designed two variants: R11^ΔLys^ with complete substitution of all 10 lysines with arginines, and R11^Lite^ with substitution of all lysines except the three (Lys32, Lys40, and Lys59) implicated in KRAS contact (Figure 2c). Because arginine is more basic than lysine (Molina et al., 2026), this substitution increases the predicted isoelectric point of R11 from 9.26 to 10.19 and 9.69, respectively (Gasteiger et al., 2005). Both variants expressed in *E. coli* in yields comparable to native R11 (3.5 and 1.5 mg L^−1^, respectively). Strikingly, BLI analysis revealed that both variants retained nanomolar binding affinity to KRAS^WT^ and KRAS^G12D^ (Figure 2b). The retention of affinity by R11^ΔLys^ despite substitution of all 10 lysines, including the three at the binding interface, suggests that the Sso7d‐derived KRAS‐binding interface tolerates extensive remodeling of basic residues, with arginine substituting effectively for the salt‐bridge and hydrogen‐bonding roles of lysine. This finding is, to our knowledge, the most extensive K → R substitution reported for an Sso7d‐derived binder that retains target affinity and informs the engineering of lysine‐reduced binders based on this scaffold.

To enable bioconjugation, we generated C‐terminal cysteine versions of both variants (R11^ΔLys^‐Cys and R11^Lite^‐Cys). Unfortunately, R11^ΔLys^‐Cys conjugates proved poorly soluble: bioconjugation reactions with maleimide reagents in PBS (pH 6.7) reproducibly resulted in protein precipitation. R11^Lite^‐Cys, by contrast, was tractable for conjugation. We thus advanced both R11‐Cys (native) and R11^Lite^‐Cys for downstream chemical functionalization.

### 2.3 Bioconjugation and cellular delivery of R11 variants

Conjugation of R11‐Cys and R11^Lite^‐Cys with BODIPY–maleimide was achieved using standard thiol–maleimide chemistry under mild reducing conditions, and conjugates were characterized by ESI Q‐TOF mass spectrometry (Figures 3 and S1).

**FIGURE 3:**
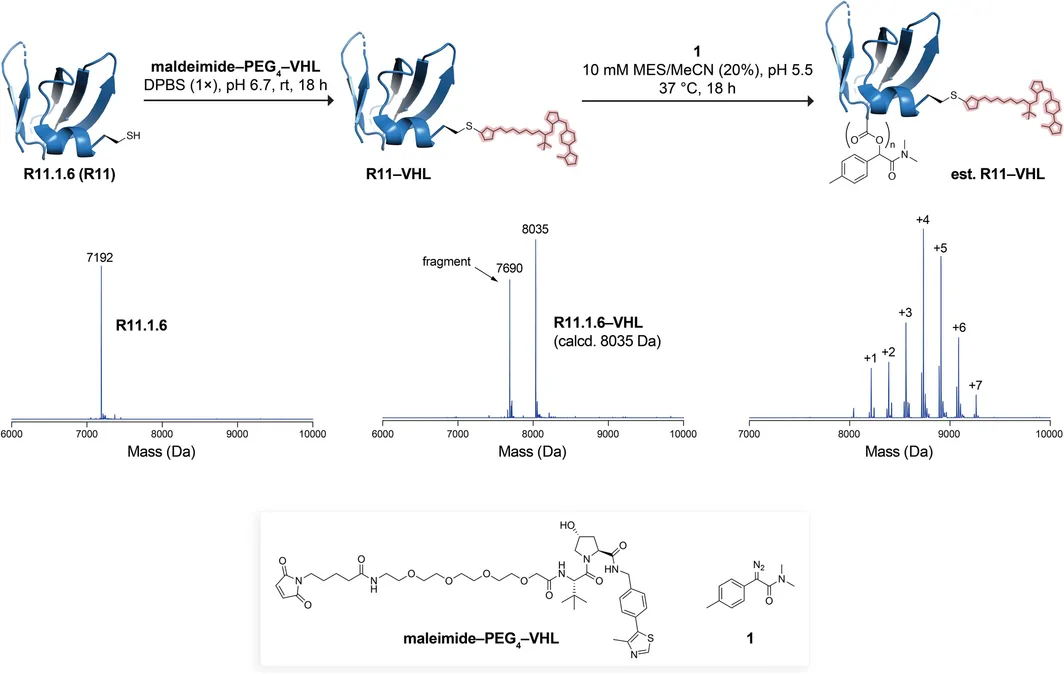
Preparation of R11–VHL and its esterification with diazo compound **1**. Esterified R11–VHL with 4–5 labels produced the most consistent results in the protein degradation studies.

To enable cellular delivery, we esterified the protein conjugates with α‐aryl‐α‐diazoamide **1**, a method previously developed by our laboratory for the cytosolic delivery of proteins (Mix et al., 2017; Okon et al., 2025; Ressler et al., 2019). Esterification of R11–BODIPY yielded conjugates bearing a median of 1–5 alkyl ester labels per protein (Figure S2). Live‐cell confocal microscopy showed intracellular BODIPY fluorescence after treatment of CHO‐K1 cells with esterified R11–BODIPY, consistent with cellular uptake of the labeled protein (Figure S3). Notably, R11^Lite^–BODIPY accumulated within SW48^G12D^ cells even without prior esterification (Figure S4), consistent with previous reports that arginine‐rich surface patches can confer cell permeability to small proteins (Chen & Pei, 2020; Fuchs & Raines, 2007). This serendipitous finding suggests that the K → R substitutions in R11^Lite^ confer a degree of intrinsic cell permeability, an additional benefit of the ubiquitination‐resistant design.

### 2.4 VHL‐dependent reduction of pan‐RAS by esterified R11–VHL

We next synthesized a maleimide–PEG_4_–VHL ligand reagent (**4**) for site‐specific cysteine modification (Scheme S1). We tested whether esterified R11–VHL (est‐R11–VHL) could induce loss of RAS proteins in cells expressing KRAS^G12D^. Cells were treated with a concentration series of est‐R11–VHL (0.5–10.0 μM) for 4 h in serum‐free media, followed by 20 h in complete media; lysates were analyzed by immunoblotting for KRAS^G12D^ and pan‐RAS (Scheme S2).

In HPAF‐II cells, est‐R11–VHL induced a dose‐dependent reduction in pan‐RAS levels, reaching ~40% loss at 10.0 μM relative to both no‐protein and unesterified R11–VHL controls (Figures 4a and S7). KRAS^G12D^ levels in the same cells trended downward but did not reach statistical significance. The preferential reduction of pan‐RAS over KRAS^G12D^ may reflect the relative cellular abundance of RAS isoforms: prior estimates suggest that non‐G12D RAS isoforms in HPAF‐II cells (~1.3 μM) substantially exceed KRAS^G12D^ levels (Lim et al., 2021), and the parent R11 protein retains nanomolar affinity for KRAS^WT^ (Figure 2b) (Kauke et al., 2017). The conjugate is thus expected to engage multiple RAS isoforms, consistent with the observed pan‐RAS preference.

**FIGURE 4:**
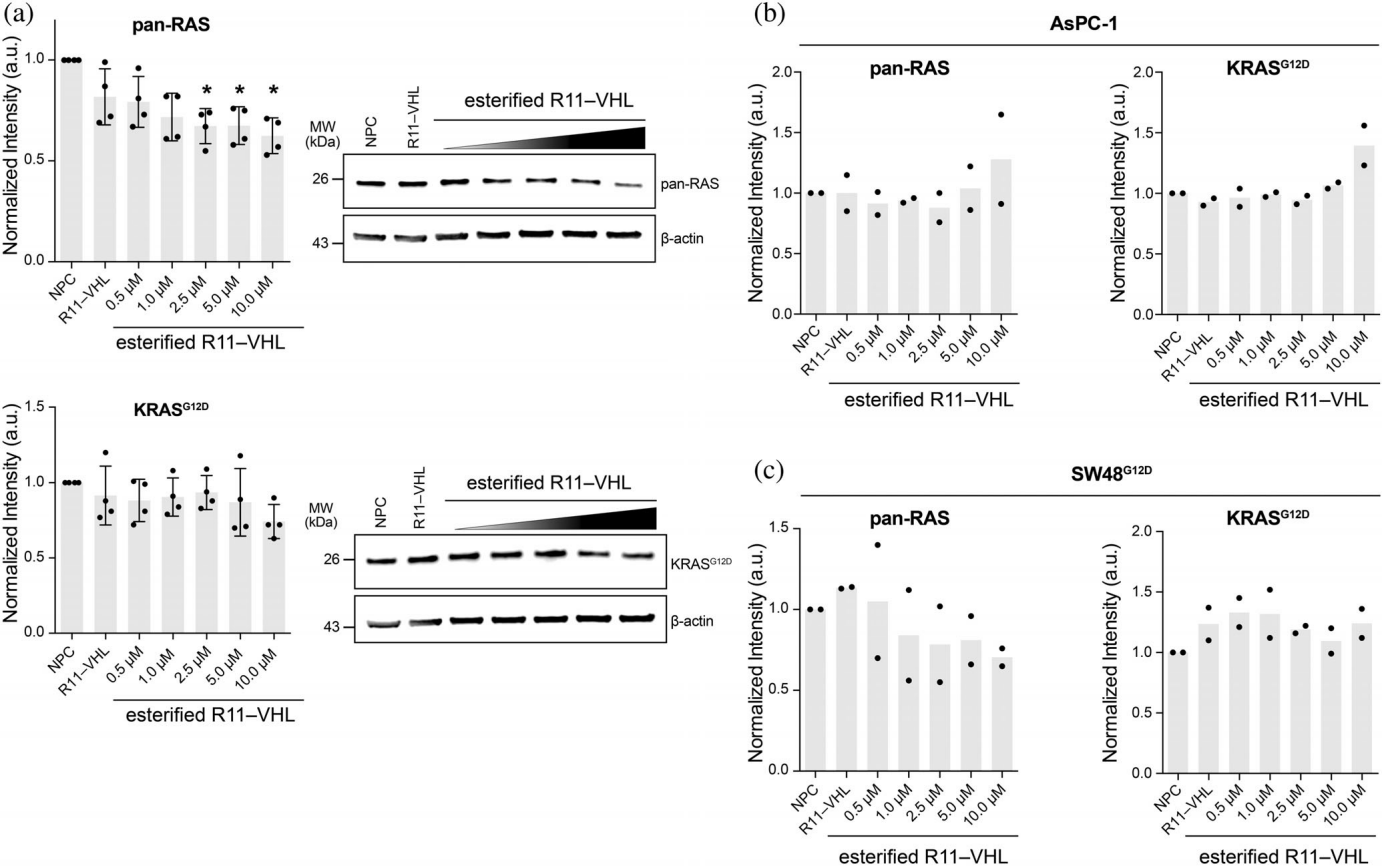
Esterified R11–VHL induces dose‐dependent reduction of pan‐RAS in HPAF‐II cells but is inactive in two additional KRAS^G12D^‐expressing cell lines. (a) Quantification of KRAS^G12D^ and pan‐RAS levels in HPAF‐II cells treated with the indicated concentrations of esterified R11–VHL for 24 h, normalized to β‐Actin and to the no‐protein control (NPC). Values represent mean ± SEM from two independent biological replicates; complete immunoblots are shown in Figures S7 and S8. One‐way ANOVA, *p < 0.05. (b,c) Equivalent quantification in AsPC‐1 cells (b) and SW48^G12D^ cells (c) from a single biological replicate, included for completeness; complete immunoblots are shown in Figures S9–S12.

In two additional KRAS^G12D^‐expressing cell lines, AsPC‐1 and SW48^G12D^, we observed no reduction in pan‐RAS or KRAS^G12D^ following est‐R11–VHL treatment (Figures 4b,c and S9–S12). In both lines, KRAS^G12D^ levels trended modestly upward upon treatment, possibly reflecting compensatory upregulation as previously reported for KRAS‐targeting interventions (Kauke et al., 2018). The cell‐line dependence of degradation efficacy is consistent with prior reports of context‐dependent outcomes for protein‐based RAS degraders (Lim et al., 2021). We note that we cannot at present distinguish whether differential outcomes reflect differences in protein uptake, intracellular esterase activity, VHL expression, or RAS isoform composition; a systematic dissection of these variables is beyond the scope of this proof‐of‐concept study.

To test whether the observed pan‐RAS reduction in HPAF‐II cells is mediated by the VHL E3 ubiquitin ligase, we performed a competition experiment with free VHL ligand. Co‐treatment of HPAF‐II cells with est‐R11–VHL (10.0 μM) and excess free VHL ligand (200 μM) abolished the pan‐RAS reduction observed with est‐R11–VHL alone (Figures 5 and S13). This rescue supports a VHL‐ligand‐dependent mechanism for pan‐RAS loss and argues against a nonspecific effect of esterified‐protein delivery under these conditions. Co‐treatment was performed with a free VHL ligand rather than with proteasome or neddylation inhibitors because perturbation of ubiquitin homeostasis can affect endosomal trafficking and thereby confound interpretation (van Kerkhof et al., 2001). Together with the lack of activity of unesterified R11–VHL (Figure 4a), the competition result is consistent with a model in which esterified R11–VHL engages the VHL degradation machinery after cellular delivery.

**FIGURE 5:**
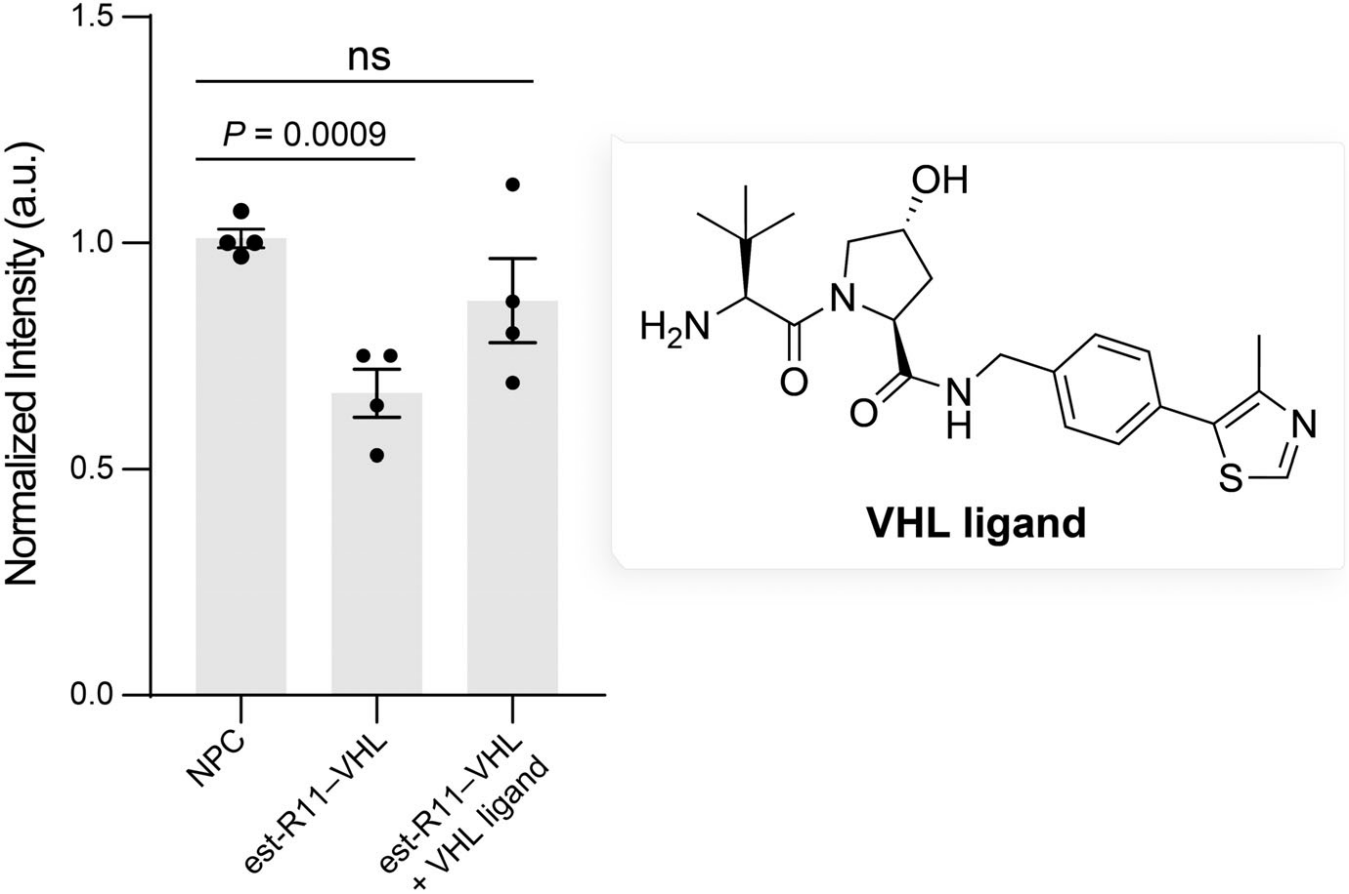
Free VHL ligand rescues pan‐RAS loss, supporting VHL‐ligand‐dependent activity of the chimeric degrader. Quantification of pan‐RAS levels in HPAF‐II cells co‐treated with esterified R11–VHL (10.0 μM) and free VHL ligand (200 μM) for 24 h, compared to esterified R11–VHL alone and to the no‐protein control (NPC). Values represent mean ± SEM from two independent biological replicates. Comparison by two‐tailed unpaired Student's *t*‐test; ns, not significant. Complete immunoblots are shown in Figure S13.

### 2.5 R11^Lite^ –VHL as a candidate ubiquitination‐resistant degrader

To assess whether the lysine‐reduced R11^Lite^ scaffold could function as a degrader, we conjugated R11^Lite^‐Cys to the maleimide–PEG_4_–VHL reagent (Figure S6) and treated HPAF‐II cells with the resulting R11^Lite^–VHL conjugate. Because R11^Lite^–BODIPY showed apparent cellular uptake without esterification (vide supra), we tested R11^Lite^–VHL directly. Although pan‐RAS reduction was observed in one replicate, the result was not reproduced in a second replicate (Figures S14 and S15). Owing to this irreproducibility, we did not pursue further quantitative characterization of R11^Lite^–VHL as a degrader. We include these results to document the behavior of the variant and to highlight that engineering an arginine‐enriched, ubiquitination‐resistant binder is not, on its own, sufficient to produce a robust degrader; additional optimization of conjugation site, linker, or delivery format is likely required.

## 3 DISCUSSION

### 3.1 Engineering insights from the R11 scaffold

The work presented here establishes several practical design principles for adapting Sso7d‐derived binders and, by extension, other small protein scaffolds, for use as chemically conjugatable degrader components.

First, the site of cysteine introduction materially affects both expression yield and target affinity. For R11, an N‐terminal cysteine yielded poorly expressed, impure protein, whereas a C‐terminal cysteine produced a robust expression construct but reduced KRASG12D affinity by 10‐fold. Designers of similar scaffolds should anticipate this trade‐off and may benefit from exploring internal conjugation handles (e.g., non‐canonical amino acids or surface‐exposed loops distal to the binding interface) when scaffold geometry permits.

Second, the Sso7d‐based KRAS‐binding interface tolerates extensive lysine‐to‐arginine substitution. Replacement of all ten lysines in R11 with arginines, including Lys32, Lys40, and Lys59, which form direct contacts with KRAS in the co‐crystal structure (Kauke et al., 2017), preserved nanomolar affinity for both KRAS^WT^ and KRAS^G12D^. This finding is significant for two reasons. From a mechanistic perspective, it implies that arginine can functionally substitute for lysine at the R11–KRAS interface, presumably by maintaining the salt‐bridging and hydrogen‐bonding interactions originally provided by the lysine ε‐amine. From a practical perspective, it demonstrates that the Sso7d scaffold is unusually tolerant of K → R remodeling, offering a straightforward route to ubiquitination‐resistant variants of this widely used binder family (Koide et al., 1998; Luo et al., 2022).

Third, complete K → R substitution carries a solubility cost in the conjugation context. Although R11^ΔLys^ itself expressed and folded normally, its C‐terminal cysteine variant precipitated upon maleimide conjugation. This insolubility suggests that for fully arginine‐substituted binders, conjugation conditions or linker chemistries may need to be adapted, for example, by using more polar linkers, solubilizing tags, or excipients (Cheah et al., 2022). The lysine‐lite design (R11^Lite^), which preserves three lysines at the binding interface, avoids this solubility liability while still substantially reducing the available ubiquitination sites.

Fourth, arginine enrichment may confer intrinsic cell permeability. R11^Lite^–BODIPY accumulated in SW48^G12D^ cells without esterification, consistent with the well‐documented capacity of arginine‐rich surface patches to mediate cellular uptake (Chen & Pei, 2020; Fuchs & Raines, 2007). While we did not pursue quantitative comparisons of uptake efficiency between native R11 and R11^Lite^, this observation suggests that the K → R substitution strategy could serve a dual purpose: reducing ubiquitination susceptibility while simultaneously enhancing cellular delivery. Whether this property is generalizable to other Sso7d‐derived binders, or to other small scaffolds with similar surface lysine distributions, remains an open question worthy of future investigation.

### 3.2 Functional outcomes and their interpretation

The functional data presented here provide proof of concept for an exogenously delivered chimeric degrader and also define the limits of this initial design. In HPAF‐II cells, esterified R11–VHL produced a reproducible, dose‐dependent, and VHL‐dependent reduction in pan‐RAS levels. The VHL ligand competition experiment (Figure 5) is particularly informative: it supports a requirement for VHL‐ligand‐dependent engagement and argues against nonspecific esterified‐protein delivery as the sole cause of pan‐RAS loss.

Three limitations of the current design merit explicit discussion. First, the degradation effect is modest (~40% reduction at 10 μM) and is not selective for KRAS^G12D^ over other RAS isoforms. The latter is unsurprising given that R11 retains nanomolar affinity for KRAS^WT^ (Kauke et al., 2017) and that non‐G12D RAS isoforms substantially outnumber KRAS^G12D^ in HPAF‐II cells. A truly selective KRAS^G12D^ degrader will likely require either a more selective binder or a degrader format that exploits kinetic selectivity. Second, degradation is cell‐line dependent, with no detectable activity in AsPC‐1 or SW48^G12D^ cells. We cannot at present attribute this variability to a single cause; differences in cellular uptake, intracellular esterase activity, VHL ligase abundance, proteasome activity differences, RAS isoform composition, and compensatory upregulation pathways (Kauke et al., 2018) may all contribute. Resolving these factors will require systematic characterization across cell lines, which we identify as an important direction for future work. Third, the conjugate itself may be susceptible to self‐ubiquitination or other intracellular loss mechanisms, a possibility consistent with the lack of additional pan‐RAS loss upon extended incubation, though this has not been established directly here. Although the R11^Lite^ design partially addresses this concern by reducing the number of available ubiquitination sites, the resulting conjugate did not reproducibly degrade in cells (Figure S15), indicating that ubiquitination resistance alone is insufficient.

### 3.3 Comparison with existing biodegrader strategies

The chimeric degrader strategy described here occupies a conceptual middle ground between small‐molecule PROTACs and protein‐based bioPROTACs. Like small‐molecule PROTACs (Bondeson et al., 2015; Sakamoto et al., 2001), our conjugate uses a chemical E3 ligase recruiter and is delivered exogenously rather than expressed intracellularly. Like bioPROTACs (Lim et al., 2020; Lim et al., 2021; Teng et al., 2021; VanDyke et al., 2022), it exploits the affinity and selectivity of an evolved protein binder, enabling targeting of proteins lacking suitable small‐molecule binding pockets. This hybrid format may be particularly useful for rapid prototyping of degraders against undruggable targets for which protein binders are available, but small‐molecule POI ligands are not.

Our approach is also complementary to microinjection‐based exogenous delivery, which has recently enabled a detailed comparison of degradation rates across diverse E3 recruitment domains in single cells (Vukovic et al., 2026). Microinjection provides precise control over intracellular concentration and a defined initiation time for kinetic analysis, while chemical delivery via esterification or cell‐penetrating sequences enables application to larger cell populations and to formats less amenable to physical injection. The trade‐off is that esterification is a stochastic modification that produces a distribution of labeling states and depends on intracellular esterase activity for unmasking. Future iterations may benefit from combining the R11 engineering presented here with alternative delivery strategies, including cell‐penetrating peptide conjugation (Okon et al., 2024; Peitz et al., 2002; Schwarze et al., 1999; Shinga et al., 2022) and cell‐permeant miniature protein vectors (Wissner et al., 2018), to achieve higher and more uniform intracellular concentrations.

### 3.4 Outlook

The work described here provides a foundation for further development of Sso7d‐derived chimeric degraders. Three directions appear especially promising. First, alternative conjugation sites (e.g., internal non‐canonical amino acid handles or solvent‐exposed loops distal to the binding interface) could preserve the full nanomolar affinity of R11 for KRAS^G12D^ while still enabling site‐specific functionalization. Second, linker optimization, including length, polarity, and cleavable elements, may improve ternary‐complex geometry and degradation efficiency. Third, alternative E3 ligase recruiters (e.g., cereblon, IAP, or RNF114 ligands) should be tested in this format, as the optimal POI–E3 pairing is generally unpredictable a priori and may differ across distinct RAS isoforms or cellular contexts (Lim et al., 2021).

More broadly, our results show that protein engineering, particularly adapting binder scaffolds for chemical conjugation, intracellular use, and reduced susceptibility to lysine‐dependent self‐modification, is a critical but underexplored component of protein‐based degrader development. As the field moves toward exogenously deliverable protein degraders, scaffold engineering of the type described here is likely to be a major determinant of success, alongside advances in delivery chemistry and E3 ligase recruitment.

## 4 EXPERIMENTAL PROCEDURES

### 4.1 Materials and reagents

All commercially sourced reagents were used as supplied without further purification. *tert*‐Butyl 14‐hydroxy‐3,6,9,12‐tetraoxatetradecanoate, *p*‐toluenesulfonyl chloride, *N*,*N*‐diisopropylethylamine (DIPEA), triethylamine, sodium azide, 5,5′‐dithio‐bis‐(2‐nitrobenzoic acid) (DTNB, Ellman's reagent), and tris(2‐carboxyethyl)phosphine hydrochloride (TCEP) were from Sigma–Aldrich (Milwaukee, WI). 5‐(2,5‐Dioxo‐2,5‐dihydro‐1*H*‐pyrrol‐1‐yl)pentanoic acid and the VHL ligand were from AmBeed (Arlington Heights, IL). Palladium on carbon (10% w/w) was from TCI America (Portland, OR). 1‐[Bis(dimethylamino)methylene]‐1*H*‐1,2,3‐triazolo[4,5‐*b*]pyridinium 3‐oxide hexafluorophosphate (HATU) was from Chem‐Impex International (Wood Dale, IL). BODIPY FL maleimide was from Thermo Fisher Scientific (Waltham, MA). Trifluoroacetic acid was from Beantown Chemical (Hudson, NH). PD‐10 desalting columns and PD MiniTrap G‐25 columns were from Cytiva (Buckinghamshire, UK). Amicon Ultra 0.5‐mL centrifugal filters (3‐kDa MWCO) were from Sigma–Aldrich. α‐Aryl‐α‐diazoamide reagent **1** was synthesized as described previously (Mix et al., 2017).

Dulbecco's phosphate‐buffered saline (DPBS) without Ca^2+^/Mg^2+^ (Gibco, product 14190144) was used for procedures involving mammalian cells. Anhydrous solvents were obtained from an in‐house Glass Contour Solvent System (SG Water, Nashua, NH). Water was purified using a Milli‐Q IQ 7000 system (Sigma–Aldrich) and used for all aqueous buffers.

### 4.2 Instrumentation

^1^H and ^13^C NMR spectra were recorded on a Bruker Avance Neo 400 MHz or Bruker Avance 500 MHz spectrometer; chemical shifts (*δ*, ppm) are reported relative to residual undeuterated solvent peaks. All ^1^H NMR data are represented as chemical shift, multiplicity (s, singlet; d, doublet; q, quartet; h, quintet; and m, multiplet), coupling constant (*J*, Hz), and integration. High‐resolution mass spectra were obtained with an AccuTOF‐DART 4G from JEOL (Peabody, MA) by helium DART ionization.

Protein ESI mass spectrometry was performed on a 6530 Accurate‐Mass Q‐TOF LC/MS (Agilent Technologies) equipped with a PLRP‐S column (1000 Å pore size, 5‐μm particle size, 50 mm × 2.1 mm; Agilent Technologies) using a gradient of 5%–95% v/v acetonitrile (0.1% v/v formic acid) in water (0.1% v/v formic acid) over 7 min. Protein concentrations were determined by a Pierce bicinchoninic acid (BCA) assay (Thermo Fisher Scientific) on a TECAN te‐cool microplate reader (Zürich, Switzerland) or by *A*_280 nm_ from a NanoDrop 2000c spectrophotometer (Thermo Fisher Scientific). Bio‐layer interferometry was performed on a ForteBio Octet RED96 system (Sartorius). Protein purification was performed on an ÄKTA Pure FPLC system (GE Healthcare Life Sciences; Piscataway, NJ) controlled by UNICORN software. SDS–PAGE was performed using Any kD Mini‐PROTEAN TGX precast gels in a Mini‐PROTEAN Tetra cell (Bio‐Rad). Immunoblotting transfers were performed using the iBlot 2 dry blotting system (Invitrogen). Immunoblot images were acquired on an Amersham Imager 600 (GE Life Sciences). Confocal microscopy was performed on an Olympus FV1200 scanning confocal microscope. Image analysis was performed with ImageJ.

### 4.3 Conditions

All procedures were performed at ambient temperature (~23°C) and pressure (1.0 atm) unless indicated otherwise. The phrase “concentrated under reduced pressure” refers to the removal of solvents and other volatile materials using a rotary evaporator at water aspirator pressure (<20 Torr) while maintaining the water‐bath temperature at 30–40°C. Residual solvent was removed from samples by the vacuum (<0.1 Torr) achieved by a mechanical belt‐drive oil pump.

### 4.4 Synthesis of the maleimide–PEG_4_ –VHL reagent (**4**)

The route for the synthesis of maleimide–PEG_4_–VHL (**4**) is shown in Scheme S1.

tert*‐Butyl 14‐azido‐3,6,9,12‐tetraoxatetradecanoate (**2**)*. To a solution of *tert*‐butyl 14‐hydroxy‐3,6,9,12‐tetraoxatetradecanoate (400 mg, 1.30 mmol) in anhydrous CH_2_Cl_2_ (5 mL) at 0°C was added *p*‐toluenesulfonyl chloride (247 mg, 1.30 mmol) and triethylamine (0.27 mL, 1.95 mmol). The reaction mixture was stirred for 18 h, then washed with water (3×), sat. aq. NaHCO_3_, water, and brine. The organic layer was dried over Na_2_SO_4_(s), filtered, and concentrated under reduced pressure. The crude tosylate was dissolved in anhydrous DMF (5 mL); sodium azide (127 mg, 1.95 mmol) was added, and the mixture was stirred at 60°C for 18 h. DMF was removed by azeotropic distillation with toluene under reduced pressure. The residue was partitioned between water and ethyl acetate; the organic extract was dried over Na_2_SO_4_(s), filtered, concentrated, and purified by silica gel chromatography (hexanes/ethyl acetate) to afford compound **2** (399 mg, 92% over two steps). ^1^H NMR (500 MHz, CDCl_3_, *δ*): 4.00 (s, 2H), 3.73–3.62 (m, 14H), 3.36 (s, 2H), 1.46 (s, 9H). ^13^C{^1^H} NMR (500 MHz, CDCl_3_, *δ*): 169.64, 169.63, 81.50, 77.37, 77.11, 76.86, 70.69, 70.66, 70.63, 70.61, 70.58, 70.57, 70.01, 69.04, 69.01, 68.94, 50.66, 28.08. HRMS (DART‐TOF) *m*/*z*: [M + H]^+^ calculated for C_14_H_27_N_3_O_6_, 334.1978; found, 334.1939.

*tert‐Butyl 20‐(2,5‐dioxo‐2,5‐dihydro‐1H‐pyrrol‐1‐yl)‐16‐oxo‐3,6,9,12‐tetraoxa‐15‐azaicosanoate (**3**)*. Azide **2** (194 mg, 0.582 mmol) was dissolved in ethanol (2.5 mL), and Pd/C (10%, 19.4 mg) was added. The mixture was stirred under H_2_(g) for 18 h, then filtered through Celite 545 and concentrated under reduced pressure to provide the corresponding amine, which was used without further purification.

5‐(2,5‐Dioxo‐2,5‐dihydro‐1*H*‐pyrrol‐1‐yl)pentanoic acid (30 mg, 0.157 mmol) was dissolved in anhydrous DMF (5.7 mL); HATU (65 mg, 0.204 mmol) and triethylamine (44 μL, 0.314 mmol) were added, and the mixture was stirred for 15 min. The amine (58 mg, 0.188 mmol), dissolved in anhydrous DMF (0.2 mL), was then added, and the mixture was stirred for 18 h. The reaction mixture was concentrated under reduced pressure, partitioned between ethyl acetate and 1.0 N HCl, and washed with water, sat. aq. NaHCO_3_, water, and brine. The organic layer was dried over Na_2_SO_4_(s), filtered, concentrated, and purified by silica gel chromatography (CH_2_Cl_2_/MeOH, 0–5% v/v MeOH) to give compound **3** (48.6 mg, 64%). ^1^H NMR (500 MHz, CDCl_3_, *δ*): 7.28 (s, 1H), 6.70 (s, 1H), 6.12 (s, 1H), 4.03 (s, 2H), 3.74–3.62 (m, 12H), 3.58–3.54 (m, 4H), 3.46–3.43 (m, 2H), 2.23 (d, *J* = 7.0 Hz, 2H), 1.67–1.62 (m, 4H), 1.49 (s, 9H). ^13^C{^1^H} NMR (500 MHz, CDCl_3_, *δ*): 172.41, 170.82, 169.64, 134.14, 134.12, 134.09, 134.04, 81.60, 77.30, 77.04, 76.79, 70.72, 70.61, 70.58, 70.54, 70.52, 70.26, 69.89, 69.01, 39.19, 37.33, 35.80, 28.12, 28.05, 22.71. HRMS (DART‐TOF) *m*/*z*: [M + H]^+^ calculated for C_23_H_38_N_2_O_9_, 487.2650; found, 487.2105.

*Maleimide–PEG*_*4*_ *–VHL conjugate (**4**)*. Compound **3** (44 mg, 0.0904 mmol) was dissolved in CH_2_Cl_2_ (0.6 mL) and cooled to 0°C. Trifluoroacetic acid (0.15 mL) was added, and the reaction mixture was stirred at 0°C for 2.5 h. Solvents were removed by azeotropic distillation with toluene under reduced pressure. The residue was dissolved in anhydrous DMF (2.0 mL); HATU (38 mg, 0.118 mmol) and triethylamine (38 μL, 0.271 mmol) were added, and the mixture was stirred for 15 min. VHL ligand (32 mg, 0.0753 mmol), dissolved in anhydrous DMF (0.5 mL), was then added, and the reaction mixture was stirred for 18 h. The reaction mixture was concentrated by azeotropic distillation with toluene under reduced pressure, partitioned between ethyl acetate and 1.0 N HCl, and washed with water, sat. aq. NaHCO_3_, water, and brine. The organic extract was dried over Na_2_SO_4_(s), filtered, concentrated, and purified by silica gel chromatography (CH_2_Cl_2_/MeOH, 0–10% v/v MeOH), followed by reversed‐phase chromatography (H_2_O/MeCN), to afford compound **4** (35.4 mg, 56%). ^1^H NMR (500 MHz, CDCl_3_, *δ*): 8.70 (s, 1H), 7.40 (s, 1H), 7.37 (s, 4H), 7.23 (s, 1H), 6.66 (s, 2H), 6.36 (d, *J* = 12.9 Hz, 1H), 4.74 (s, 1H), 4.61–4.50 (m, 3H), 4.37 (d, *J* = 9.6 Hz, 1H), 4.08–4.01 (m, 3H), 3.74–3.61 (m, 10H), 3.60 (s, 4H), 3.53 (h, *J* = 4.7 Hz, 4H), 3.39 (q, *J* = 5.3 Hz, 2H), 2.52 (s, 4H), 2.18–2.13 (m, 3H), 1.59 (h, *J* = 3.4 Hz, 4H), 0.96 (s, 9H). ^13^C{^1^H} NMR (500 MHz, CDCl_3_, *δ*): 172.90, 171.41, 171.03, 170.28, 150.48, 138.43, 134.23, 130.96, 129.64, 128.31, 77.41, 77.16, 76.91, 71.06, 70.91, 70.70, 70.60, 70.35, 70.24, 69.95, 58.69, 57.15, 56.96, 43.39, 39.33, 37.52, 36.30, 35.84, 35.44, 28.21, 26.56, 22.84, 16.14. HRMS (DART‐TOF) *m*/*z*: [M + H]^+^ calculated for C_41_H_58_N_6_O_11_S, 843.3957; found, 843.8133.

### 4.5 Cloning of R11 variants

Genes encoding R11.1.6, R11‐Cys, R11^ΔLys^, R11^ΔLys^‐Cys, R11^Lite^, R11^Lite^‐Cys, and Cys‐R11.1.6 were obtained as gBlocks (Integrated DNA Technologies) and cloned into the pE‐SUMO vector (Kauke et al., 2017) using the In‐Fusion HD Cloning Kit (Takara) and CloneAmp HiFi PCR Premix according to the manufacturer's instructions. PCR amplification, ligation, and bacterial transformation were performed following the New England Biolabs Q5 Site‐Directed Mutagenesis Kit (E0554) protocol. Plasmid identity was confirmed by Sanger sequencing. The amino acid sequences of R11 variants used in this study are provided in Table S1.

### 4.6 Expression and purification of R11 variants

All R11 variants were expressed as N‐terminal hexahistidine (6 × His)–SUMO fusion proteins from the pE‐SUMO vector in *E. coli* Rosetta 2 (DE3) cells, as described previously (Kauke et al., 2017). An overnight starter culture in Luria–Bertani media (LB) supplemented with kanamycin (50 μg mL^−1^) and carbenicillin (100 μg mL^−1^) was used to inoculate (1:100 v/v) fresh LB containing the same antibiotics. Cultures were grown at 37°C until OD_600 nm_ reached 0.6–0.8, at which point protein expression was induced by addition of isopropyl β‐d‐1‐thiogalactopyranoside (IPTG) to a final concentration of 1.0 mM. Cultures were transferred to 20°C and incubated overnight. Cells were harvested by centrifugation at 4300*g* for 15 min at 4°C; pellets were flash‐frozen in N_2_(l) and stored at −70°C until use.

For purification, frozen pellets were thawed on ice and resuspended in sonication buffer (which was 50 mM sodium phosphate buffer, pH 7.3, containing 300 mM NaCl, 10% v/v glycerol, and the recommended amount of complete EDTA‐free Protease Inhibitor Cocktail (Roche 11873580001); 10 mL per 500 mL of culture) and lysed by sonication. Lysates were clarified by centrifugation at 10,000*g* for 25 min at 4°C, and the supernatant was filtered through a 0.45‐μm PES membrane. Clarified lysate was loaded onto a Talon Superflow Metal Affinity Resin column (Takara 635669; 2 mL of resin per 100 mL of culture) pre‐equilibrated with 10 column volumes (CV) of wash buffer 1 (PBS, pH 7.3). The resin was washed with 15 CV of wash buffer 1, then 15 CV of wash buffer 2 (PBS, 10 mM imidazole, pH 7.3). Bound protein was eluted with 5 CV of elution buffer (PBS, 150 mM imidazole, pH 7.3).

Eluate was buffer‐exchanged into PBS (pH 7.3) and concentrated to 2–5 mg mL^−1^ using an Amicon Ultra centrifugal filter (3‐kDa MWCO). The 6 × His–SUMO tag was removed by overnight digestion at 4°C with SUMO protease 1 (Ulp1). The digest was reapplied to a fresh Talon column pre‐equilibrated with wash buffer 1; the flow‐through containing the cleaved R11 variant was collected and reapplied twice more to ensure complete removal of His‐tagged species. The final flow‐through was buffer‐exchanged into PBS (pH 7.3), concentrated to 2–5 mg mL^−1^, flash‐frozen in N_2_(l), and stored at −70°C until use.

For purification of R11 variants containing a C‐terminal cysteine, the cysteine thiol was protected throughout purification by including DTNB (1 mM, adjusted to pH 7.3) in all buffers, generating the 2‐nitro‐5‐thiobenzoate (NTB)‐protected species. Protein purity was assessed by SDS–PAGE; identity was confirmed by ESI Q‐TOF mass spectrometry.

### 4.7 Expression and purification of KRAS proteins

The G‐domains (residues 1–166) of human KRAS^WT^ and KRAS^G12D^ (isoform 4B) were expressed and purified as described previously (Kauke et al., 2017). Briefly, each protein was expressed as an N‐terminal 6 × His‐tagged fusion in BL21(DE3) *E. coli*. We compared 6 × His–SUMO–KRAS and 6 × His–KRAS constructs and observed higher yields of size‐exclusion‐purified, GppNHp‐loaded protein from the latter (6 mg L^−1^ vs. 2 mg L^−1^, accounting only for the molecular weight of KRAS). Following purification, both proteins were loaded with the non‐hydrolyzable GTP analog GppNHp and biotinylated with EZ‐Link Sulfo‐NHS‐LC‐Biotin (Life Technologies) as described previously (Kauke et al., 2017). Biotinylated, GppNHp‐loaded KRAS proteins were supplemented with glycerol (10% v/v final), flash‐frozen in N_2_(l), and stored at −80°C until use.

### 4.8 Bio‐layer interferometry

Binding affinities of R11 variants and their VHL conjugates to KRAS^WT^ and KRAS^G12D^ were measured by bio‐layer interferometry (BLI) on a ForteBio Octet RED96 instrument. Assays were performed in RAS stabilization buffer (Kauke et al., 2017) supplemented with BSA (0.1% w/v), Tween‐20 (20 μL L^−1^), and GppNHp (10 μM). Biotinylated, GppNHp‐loaded KRAS^WT^ or KRAS^G12D^ was immobilized onto streptavidin‐coated BLI tips (Sartorius 18‐5020). Association was measured at a 1:3 dilution series of analyte (200 nM to 0.82 nM); dissociation was measured in buffer alone. Each run included two reference samples: KRAS‐loaded tips with no analyte and unloaded tips with 200 nM analyte. The averaged reference signal was subtracted from each binding trace. Equilibrium dissociation constants (*K*_d_) were determined from steady‐state analysis of the binding response.

### 4.9 Bioconjugation of R11 variants

C‐terminal cysteine variants of R11 (NTB‐protected) were buffer‐exchanged into PBS (pH 6.7) and adjusted to 500 μM. The NTB protecting group was removed by addition of TCEP to 5.0 mM, immediately followed by addition of BODIPY FL maleimide or compound **4** (5 equiv., 2.5 mM) from a DMSO stock. Additional DMSO was added as needed to ensure complete dissolution of the maleimide reagent, with final DMSO content kept below 10% v/v. Reaction mixtures were flushed with N_2_(g) and incubated overnight in the dark with gentle shaking. Excess reagents and TCEP were removed by two sequential passages through PD‐10 desalting columns. Conjugates were concentrated to 2–5 mg mL^−1^ using Amicon Ultra centrifugal filters (3‐kDa MWCO).

For BODIPY‐labeled proteins, dye‐to‐protein ratios were determined from absorbance at 280 nm (protein) and 509 nm (BODIPY; *ε* = 92,000 M^−1^ cm^−1^) on a NanoDrop 2000c spectrophotometer. For VHL‐conjugated proteins, conjugation was confirmed by ESI Q‐TOF mass spectrometry.

### 4.10 Protein esterification

R11 conjugates (1.0 equiv.) were exchanged into 10 mM MES–NaOH buffer, pH 5.5, using Amicon Ultra centrifugal filters (3‐kDa MWCO). Acetonitrile and α‐aryl‐α‐diazoamide reagent **1** (100 equiv., dissolved in acetonitrile) were added such that the final acetonitrile content was 20% v/v and the final protein concentration was 120 μM in 10 mM MES–NaOH buffer, pH 5.5. Reaction mixtures were incubated overnight at 37°C with shaking. An equal volume of PBS (pH 7.4) was then added, and the mixture was clarified by passage through a 0.22‐μm Corning Costar Spin‐X centrifuge tube filter to remove precipitated protein. The filtrate was buffer‐exchanged into PBS (pH 7.4) using a PD MiniTrap G‐25 column and concentrated using an Amicon Ultra filter (3‐kDa MWCO). Final protein concentration was determined by BCA assay; esterification efficiency (median number of ester labels per protein) was determined by ESI Q‐TOF mass spectrometry.

### 4.11 Cell culture

HPAF‐II cells (ATCC) were cultured in Eagle's Minimum Essential Medium (EMEM). SW48^G12D^ cells (gift of F. White) and AsPC‐1 cells (gift of D.J. Irvine) were cultured in RPMI 1640. CHO‐K1 cells (ATCC) were cultured in F‐12K medium. All media were supplemented with 10% v/v fetal bovine serum (FBS; Life Technologies), 100 U mL^−1^ penicillin, and 100 μg mL^−1^ streptomycin (Gibco). Cells were maintained at 37°C in a humidified atmosphere of 5% v/v CO_2_. Cell lines were used between passages 5 and 12.

### 4.12 Confocal microscopy

Cells were seeded in complete medium at 50,000 cells per well in eight‐well chamber slides (Nunc Lab‐Tek II Chamber Slide System) 24 h prior to treatment. On the day of treatment, medium was aspirated and replaced with FBS‐free medium (containing 100 U mL^−1^ penicillin and 100 μg mL^−1^ streptomycin) supplemented with the indicated BODIPY‐labeled protein. Cells were incubated for 4 h at 37°C, after which the protein‐containing medium was replaced with complete medium (10% v/v FBS) for an additional 2 h at 37°C. Cells were washed twice with DPBS and stained with Hoechst 33342 (2 μg mL^−1^ in DPBS) for 5 min at 37°C. After Hoechst removal, cells were chilled on ice for 5 min, then membranes were stained with wheat germ agglutinin (WGA)–Alexa Fluor 647 (5 μg mL^−1^ in DPBS) for 15 min on ice. Cells were washed twice with ice‐cold DPBS and maintained in DPBS supplemented with 20 mM HEPES on ice until imaging.

Live cells were imaged on an Olympus FV1200 scanning confocal microscope. Excitation/emission settings were Hoechst 33342, *λ*_ex_ = 405 nm and *λ*_em_ = 450 nm; BODIPY, *λ*_ex_ = 503 nm and *λ*_em_ = 512 nm; and WGA–Alexa Fluor 647, *λ*_ex_ = 647 nm and *λ*_em_ = 700 nm. Acquisition and processing parameters were held constant across samples. Images were processed with ImageJ (Schroeder et al., 2021).

### 4.13 Cellular degradation assays and immunoblotting

Cells were seeded in complete medium at 150,000 cells per well in 12‐well plates 24 h prior to treatment. Medium was aspirated and replaced with FBS‐free medium containing penicillin (100 U mL^−1^), streptomycin (100 μg mL^−1^), and the indicated concentration of test protein (esterified R11–VHL, R11–VHL, or R11^Lite^–VHL; 0.5–10.0 μM). Cells were incubated for 4 h at 37°C, after which FBS was added directly to each well to a final concentration of 10% v/v, and incubation was continued for an additional 20 h.

Cells were lysed in ice‐cold 1× RIPA buffer (Abcam ab156034) supplemented with 1× Halt Protease Inhibitor Cocktail (Thermo Fisher Scientific 78425), incubated on ice for 30 min with periodic vortexing, and clarified by centrifugation at 20,000 *g* for 15 min at 4°C. Total protein concentration in each lysate was determined by BCA assay (Pierce). Equal amounts of total protein were resolved on Any kD Mini‐PROTEAN TGX precast gels and transferred to PVDF membranes using the iBlot 2 system (iBlot 2 PVDF Mini Stacks, Invitrogen IB24002).

Membranes were trimmed at ~35 kDa to enable parallel probing for RAS proteins (lower portion) and β‐actin (upper portion). The β‐actin portion was washed three times (5 min each) in 1× TBST and blocked in 5% w/v non‐fat dry milk in TBST for 1 h. The RAS portion was washed four times (5 min each) in 1× TBST and blocked in 5% w/v milk in TBST for 1 h. Membranes were probed overnight at 4°C with primary antibodies diluted in blocking buffer. The following day, membranes were washed (β‐actin: 3× TBST; RAS: 4× TBST) and incubated for 1 h with the appropriate secondary antibody in blocking buffer. Membranes were washed again (β‐actin: 3× TBST; RAS: 4× TBST). β‐Actin blots were probed with a fluorescently labeled secondary antibody and imaged after drying in the dark; RAS blots were developed with a chemiluminescent substrate. Both blots were imaged on an Amersham Imager 600 (GE Life Sciences). Densitometric quantification was performed in ImageJ. RAS band intensities were normalized to the corresponding β‐actin loading control and expressed relative to the no‐protein control (NPC) within each blot. The antibodies used in this study are listed in Table S2.

### 4.14 VHL ligand competition assay

HPAF‐II cells were seeded in complete EMEM (10% v/v FBS, 100 U mL^−1^ penicillin, 100 μg mL^−1^ streptomycin) at 150,000 cells per well in 12‐well plates 24 h prior to treatment. On the day of treatment, medium was aspirated and replaced with FBS‐free EMEM containing one of the following: esterified R11–VHL (20 μM); free VHL ligand (400 μM); or esterified R11–VHL (20 μM) and free VHL ligand (400 μM) in combination. After 4 h at 37°C, an equal volume of EMEM containing 20% v/v FBS (with antibiotics) was added to each well, yielding final concentrations of 10 μM esterified R11–VHL and 200 μM VHL ligand in 10% v/v FBS. Cells were incubated for an additional 20 h at 37°C, then washed twice with 1× PBS (without Ca^2+^/Mg^2+^), detached with TrypLE Express (Gibco), and pelleted. Lysate preparation, SDS–PAGE, transfer, immunoblotting, and densitometry were performed as described above.

### 4.15 Statistical analysis

Quantification of immunoblot data is presented as mean ± standard error of the mean (SEM) across independent biological replicates, with the number of replicates indicated in the corresponding figure legend. Comparisons among multiple treatment conditions were performed by one‐way analysis of variance (ANOVA) with the significance threshold set at *p* < 0.05. Pairwise comparisons (e.g., the VHL competition rescue) were performed using two‐tailed unpaired Student's *t*‐tests. Data from single biological replicates (*n* = 1) are presented descriptively without statistical inference and are explicitly identified as such in the figure legends. All statistical analyses were performed in GraphPad Prism.

## AUTHOR CONTRIBUTIONS

**Aniekan Okon:** Methodology; investigation; writing – original draft; writing – review and editing; validation; funding acquisition; visualization. **Keith M. Cheah:** Methodology; investigation; writing – original draft; writing – review and editing; validation; visualization. **Oscar J. Molina:** Methodology; investigation; writing – review and editing; validation. **Gabriel Saenz:** Writing – review and editing; validation; funding acquisition. **K. Dane Wittrup:** Conceptualization; funding acquisition; project administration; writing – review and editing; supervision. **Ronald T. Raines:** Writing – review and editing; project administration; funding acquisition; conceptualization.

## Supporting information


**Scheme S1:** Synthetic route to maleimide–PEG_4_–VHL (**4**).
**Scheme S2:** Experimental workflow for evaluating the amount of RAS proteins in cells treated with esterified R11–VHL.
**Table S1:** Amino acid sequence of R11.1.6 and its variants.
**Table S2:** Antibodies used in this work.
**Figure S1:** Preparation of R11–BODIPY and deconvoluted ESI Q‐TOF mass spectra showing R11‐Cys (left) and R11–BODIPY (right). The fragment represents the loss of a fluorine atom.
**Figure S2:** Preparation of esterified R11–BODIPY and deconvoluted ESI Q‐TOF mass spectra showing R11–BODIPY (left) and esterified R11–BODIPY (right).
**Figure S3:** Confocal microscopy images of cellular uptake of esterified R11–BODIPY in CHO‐K1 cells. Cells were treated with 8 μM esterified R11–BODIPY. Blue channel: Hoechst (nuclei), green channel: BODIPY (R11 protein), red channel: WGA‐AF‐647 (cell membrane).
**Figure S4:** Confocal microscopy images of cellular uptake of R11^Lite^–BODIPY in SW48^G12D^ cells. Cells were treated with 15 μM R11^Lite^–BODIPY. Blue channel: Hoechst (nuclei), green channel: BODIPY (R11^Lite^ protein), red channel: WGA‐AF‐647 (cell membrane).
**Figure S5:** Confocal microscopy images of cellular uptake of esterified R11–BODIPY in SW48^G12D^ cells. Cells were treated with 5 μM esterified R11–BODIPY. Blue channel: Hoechst (nuclei), green channel: BODIPY (R11 protein), red channel: WGA‐AF‐647 (cell membrane).
**Figure S6:** Preparation of R11Lite–VHL and deconvoluted ESI Q‐TOF mass spectra showing R11^Lite^‐Cys (left) and R11^Lite^–VHL (right).
**Figure S7:** Immunoblots of HPAF‐II cells treated with esterified R11–VHL. Various concentrations of esterified R11–VHL (0.5, 1.0, 2.5, 5.0, and 10.0 μM) were incubated with cells for 24 h prior to cell harvesting and cell lysis. Cells were treated with unesterified R11–VHL at 10.0 μM as a control. Levels of pan‐RAS proteins were probed using a pan‐RAS antibody. The loading control was β‐actin. These blots were used to generate the data shown in Figure 4a. NPC, no protein control.
**Figure S8:** Immunoblots of HPAF‐II cells treated with esterified R11–VHL. Various concentrations of esterified R11–VHL (0.5, 1.0, 2.5, 5.0, and 10.0 μM) were incubated with cells for 24 h prior to cell harvesting and cell lysis. Cells were treated with unesterified R11–VHL at 10.0 μM as a control. Levels of KRAS^G12D^ were detected using a KRAS^G12D^ antibody. The loading control was β‐actin. These blots were used to generate the data shown in Figure 4a. NPC, no protein control.
**Figure S9:** Immunoblots of AsPC‐1 cells treated with esterified R11–VHL. Various concentrations of esterified R11–VHL (0.5, 1.0, 2.5, 5.0, and 10.0 μM) were incubated with cells for 24 h prior to cell harvesting and cell lysis. Cells were treated with unesterified R11–VHL at 10.0 μM as a control. Levels of pan‐RAS proteins were detected using a pan‐RAS antibody. The loading control was β‐actin. These blots were used to generate the data shown in Figure 4b. NPC, no protein control.
**Figure S10:** Immunoblots of AsPC‐1 cells treated with esterified R11–VHL. Various concentrations of esterified R11–VHL (0.5, 1.0, 2.5, 5.0, and 10.0 μM) were incubated with cells for 24 h prior to cell harvesting and cell lysis. Cells were treated with unesterified R11–VHL at 10.0 μM as a control. Levels of KRAS^G12D^ were detected with a KRAS^G12D^ antibody. The loading control was β‐actin. These blots were used to generate the data shown in Figure 4b. NPC, no protein control.
**Figure S11:** Immunoblots of SW48^G12D^ cells treated with esterified R11–VHL. Various concentrations of esterified R11–VHL (0.5, 1.0, 2.5, 5.0, and 10.0 μM) were incubated with cells for 24 h prior to cell harvesting and cell lysis. Cells were treated with unesterified R11–VHL at 10.0 μM as a control. Levels of pan‐RAS proteins were detected using a pan‐RAS antibody. The loading control was β‐actin. These blots were used to generate the data shown in Figure 4c. NPC, no protein control.
**Figure S12:** Immunoblots of SW48G12D cells treated with esterified R11–VHL. Various concentrations of esterified R11–VHL (0.5, 1.0, 2.5, 5.0, and 10.0 μM) were incubated with cells for 24 h prior to cell harvesting and cell lysis. Cells were treated with unesterified R11–VHL at 10.0 μM as a control. Levels of KRAS^G12D^ were detected with a KRAS^G12D^ antibody. The loading control was β‐actin. These blots were used to generate the data shown in Figure 4c. NPC, no protein control.
**Figure S13:** Immunoblot of competition study with esterified R11–VHL and VHL ligand in HPAF‐II cells. Cells were treated with 10.0 μM R11–VHL in the presence or absence of VHL ligand (200 μM) for 24 h prior to cell harvesting and cell lysis. Levels of pan‐RAS proteins were detected using a pan‐RAS antibody. The loading control was β‐actin. These blots were used to generate the data shown in Figure 5. NPC, no protein control.
**Figure S14:** Immunoblots of HPAF‐II cells treated with R11^Lite^–VHL. Various concentrations of R11^Lite^–VHL (0.5, 1.0, 2.5, 5.0, and 10.0 μM) were incubated with cells for 24 h prior to cell harvesting and cell lysis. Levels of pan‐RAS proteins were detected using a pan‐RAS antibody. The loading control was β‐actin. These blots were used to generate the data shown in Figure S15. NPC, no protein control.
**Figure S15:** Quantification of pan‐RAS levels in HPAF‐II cells treated with the indicated concentrations of R11^Lite^–VHL for 24 h, normalized to β‐actin and to the no‐protein control (NPC).

## Data Availability

The data that support the findings of this study are available in the supplementary material of this article.
