# Supplementary material for: A designer protein binder of KRAS enables exogenous protein degradation
Source: Protein Sci. 2026 Nov 9;35(12):e70850. doi: 10.1002/pro.70850 (PMC13746262; doi:10.1002/pro.70850)

A designer protein binder of KRAS enables exogenous protein degradation

Aniekan Okon | Keith M. Cheah | Oscar J. Molina | Gabriel Saenz | K. Dane Wittrup | Ronald T. Raines

Email: witttrup@mit.edu; rtraines@mit.edu

TABLE OF CONTENTS

Table of Contents	S1
Scheme S1	S2
Scheme S2	S2
Table S1	S3
Table S2	S3
Figure S1	S4
Figure S2	S5
Figure S3	S6
Figure S4	S7
Figure S5	S7
Figure S6	S8
Figure S7	S8
Figure S8	S9
Figure S9	S10
Figure S10	S11
Figure S11	S12
Figure S12	S13
Figure S13	S14
Figure S14	S15
Figure S15	S16
NMR Spectra	S17

Scheme S1 Synthetic route to maleimide–PEG$_4$–VHL (**4**)

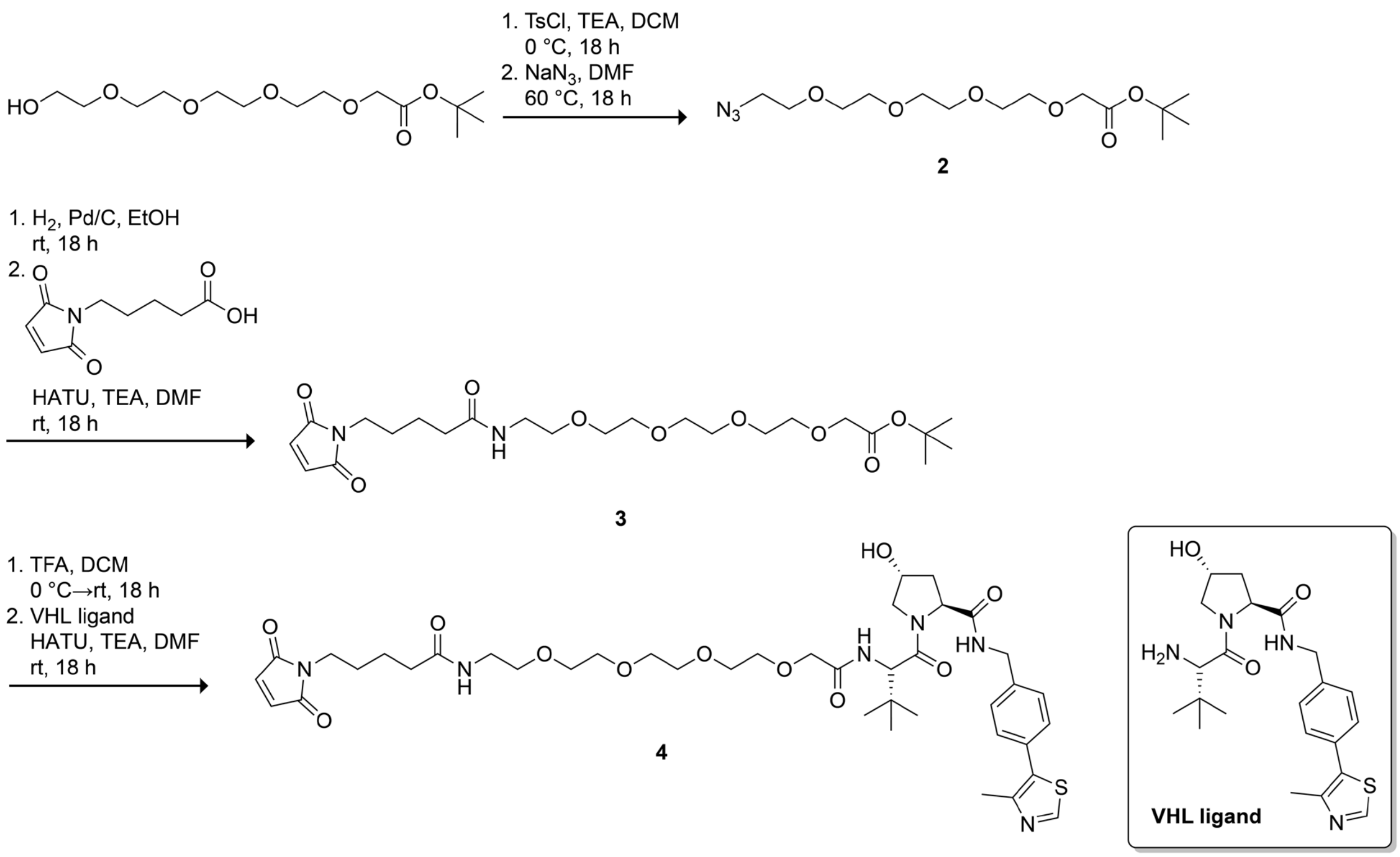

SCHEME S2 Experimental workflow for evaluating the amount of RAS proteins in cells treated with esterified R11–VHL

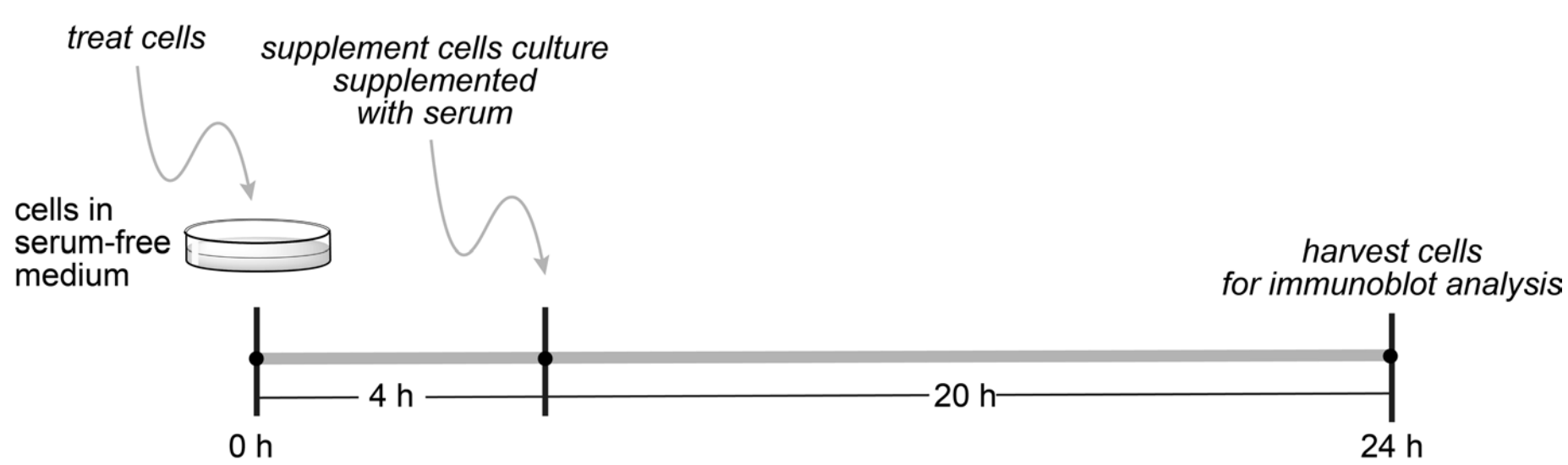

TABLE S1 Amino acid sequence of R11.1.6 and its variants

Protein	Sequence	MW (Da)
R11.1.6	ATVKFTHQGEEKQVDISKIKWVIRWGQYIWFKYDEDGGAKGWGYVSEKDAPKELLQ MLKKR	7245.33
R11-Cys	ATVKFTHQGEEKQVDISKIKWVIRWGQYIWFKYDEDGGAKGWGYVSEKDAPKELLQ MLKK**C**	7192.28
R11^{Lite}	ATVRFTHQGEERQVDISRIRWVIRWGQYIWFKYDEDGGAKGWGYVSERDAPRELLQ MLKRR	7441.42
R11^{Lite}-Cys	ATVRFTHQGEERQVDISRIRWVIRWGQYIWFKYDEDGGAKGWGYVSERDAPRELLQ MLKR**C**	7388.38
R11$^{\Delta Lys}$	ATVRFTHQGEERQVDISRIRWVIRWGQYIWFRYDEDGGARGWGYVSERDAPRELLQ MLRRR	7525.46
R11$^{\Delta Lys}$-Cys	ATVRFTHQGEERQVDISRIRWVIRWGQYIWFRYDEDGGARGWGYVSERDAPRELLQ MLRR**C**	7472.42
Cys-R11	**C**TVKFTHQGEEKQVDISKIKWVIRWGQYIWFKYDEDGGAKGWGYVSEKDAPKELLQ MLKKR	7277.39

TABLE S2 Antibodies used in this work

Antibody	Supplier (product number)	Dilution
pan-RAS (C-4)	Santa Cruz Biotechnology (sc-166691)	1:5000
RAS^{G12D} (D8H7) rabbit	Cell Signaling Technology (14429S)	1:5000
human/mouse/rat β-actin	R&D Systems (MAB8929)	1:25000
anti-mouse IgG (H+L), HRP conjugate	Promega (W402B)	1:10000
anti-rabbit IgG (H+L), HRP conjugate	Promega (W401B)	1:10000
anti-mouse IgG (H+L), DyLight™ 680 conjugate	Cell Signaling Technology (5470S)	1:4000

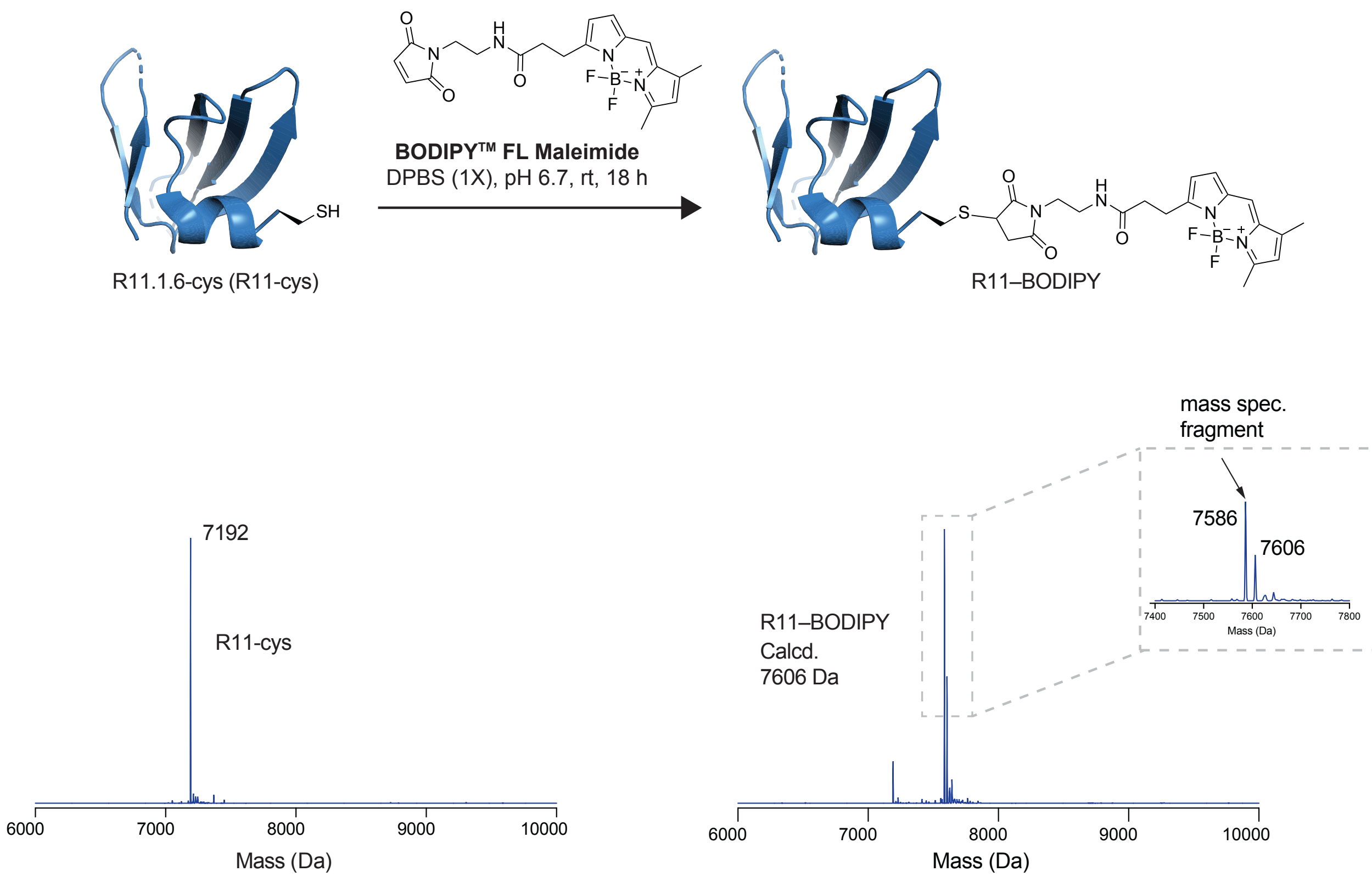

FIGURE S1 Preparation of R11–BODIPY and deconvoluted ESI Q-TOF mass spectra showing R11-Cys (left) and R11–BODIPY (right). The fragment represents the loss of a fluorine atom.

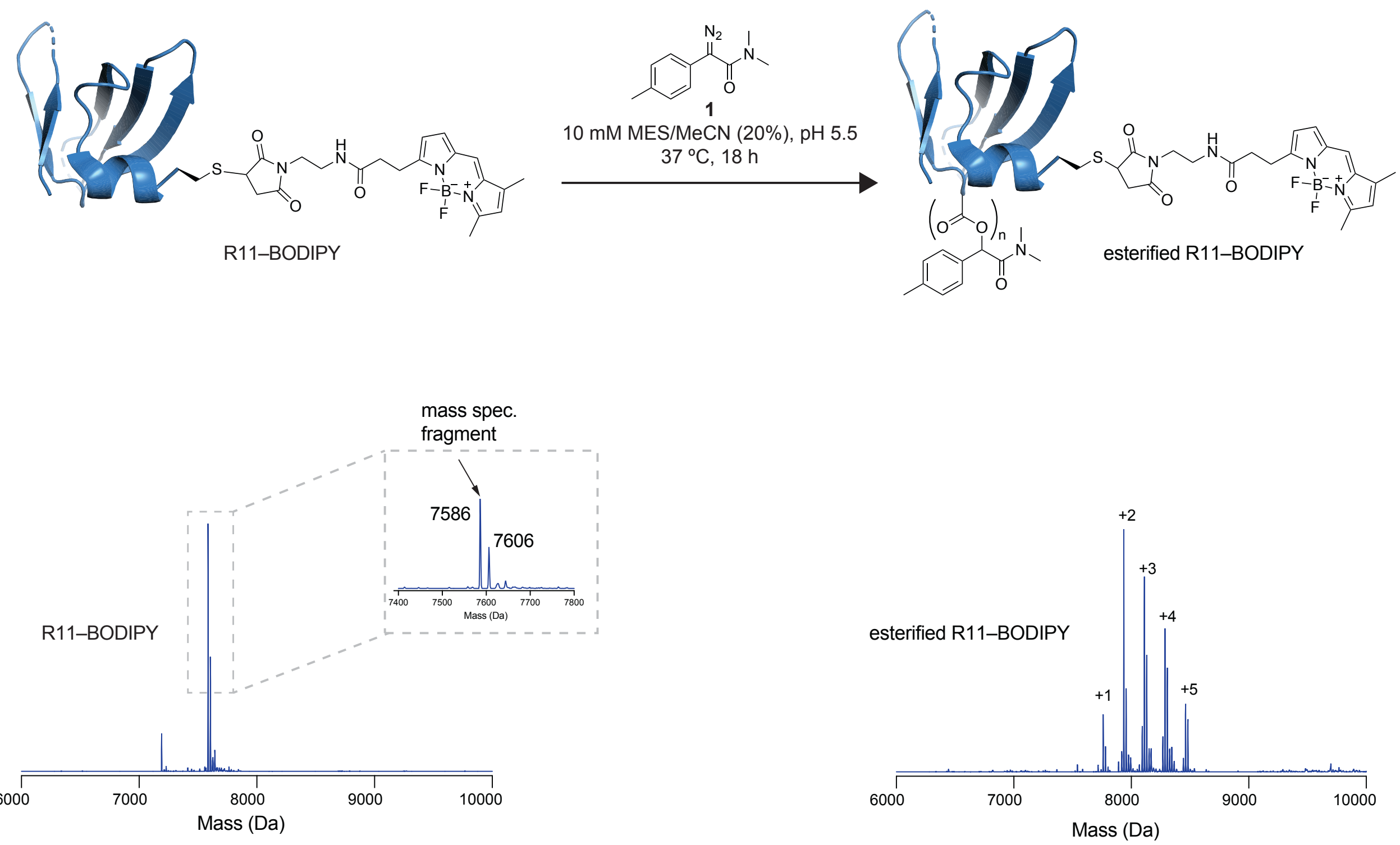

FIGURE S2 Preparation of esterified R11–BODIPY and deconvoluted ESI Q-TOF mass spectra showing R11–BODIPY (left) and esterified R11–BODIPY (right).

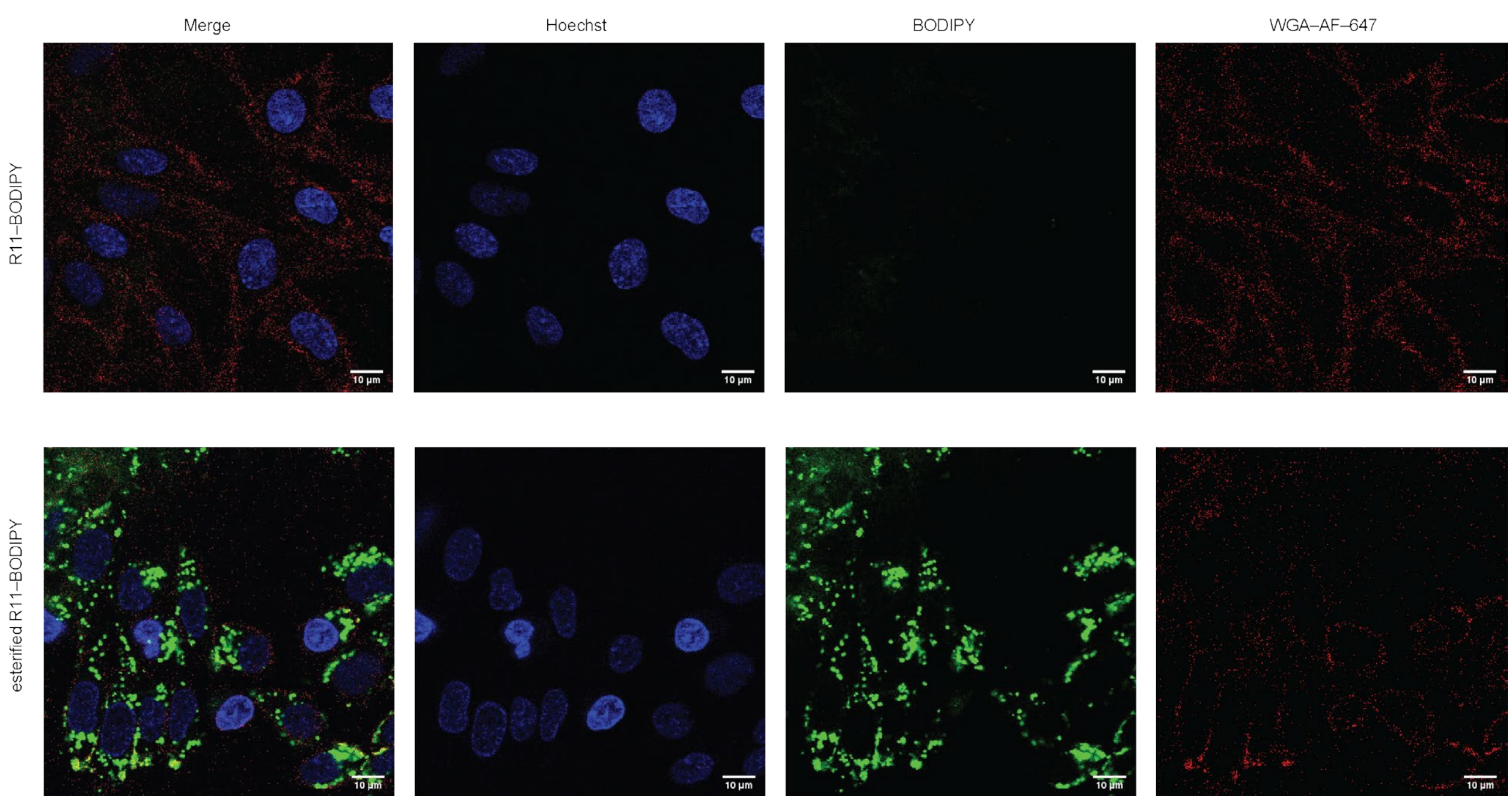

FIGURE S3 Confocal microscopy images of cellular uptake of esterified R11–BODIPY in CHO-K1 cells. Cells were treated with 8 μM esterified R11–BODIPY. Blue channel: Hoechst (nuclei), green channel: BODIPY (R11 protein), red channel: WGA-AF-647 (cell membrane).

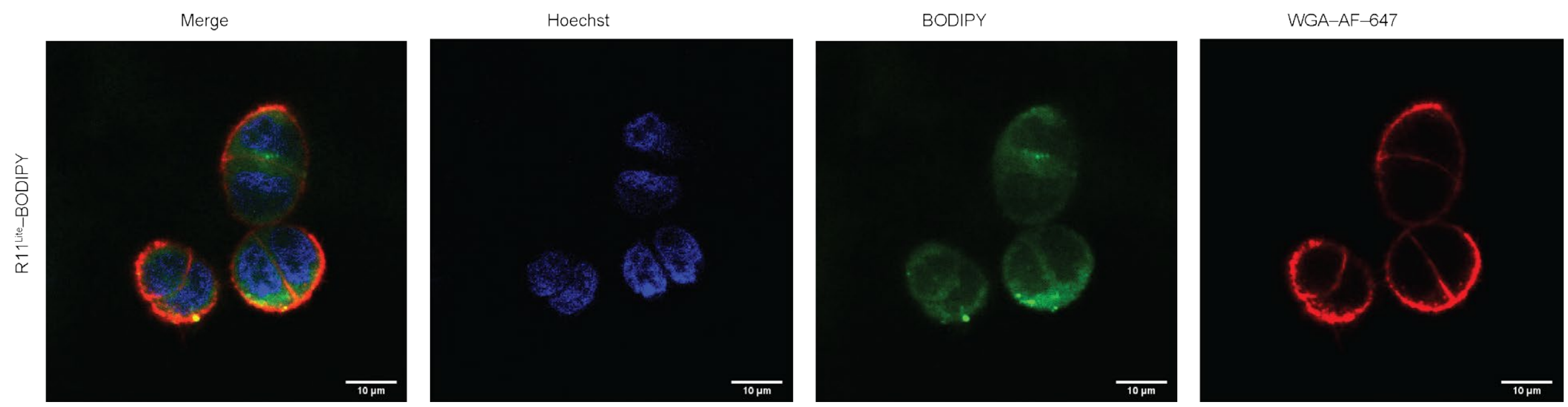

FIGURE S4 Confocal microscopy images of cellular uptake of R11^{Lite}–BODIPY in SW48^{G12D} cells. Cells were treated with 15 μM R11^{Lite}–BODIPY. Blue channel: Hoechst (nuclei), green channel: BODIPY (R11^{Lite} protein), red channel: WGA-AF-647 (cell membrane).

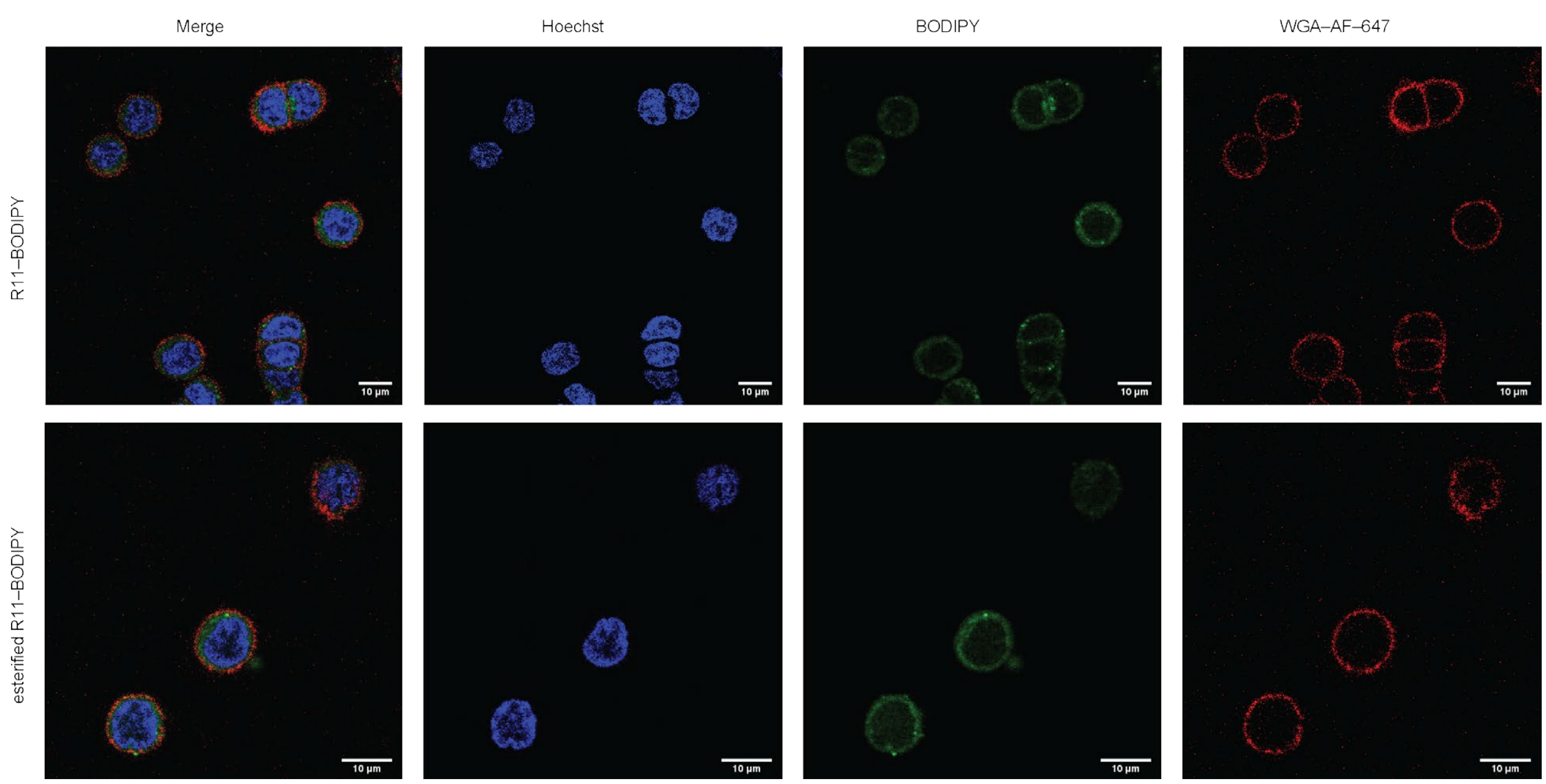

FIGURE S5 Confocal microscopy images of cellular uptake of esterified R11–BODIPY in SW48^{G12D} cells. Cells were treated with 5 μM esterified R11–BODIPY. Blue channel: Hoechst (nuclei), green channel: BODIPY (R11 protein), red channel: WGA-AF-647 (cell membrane).

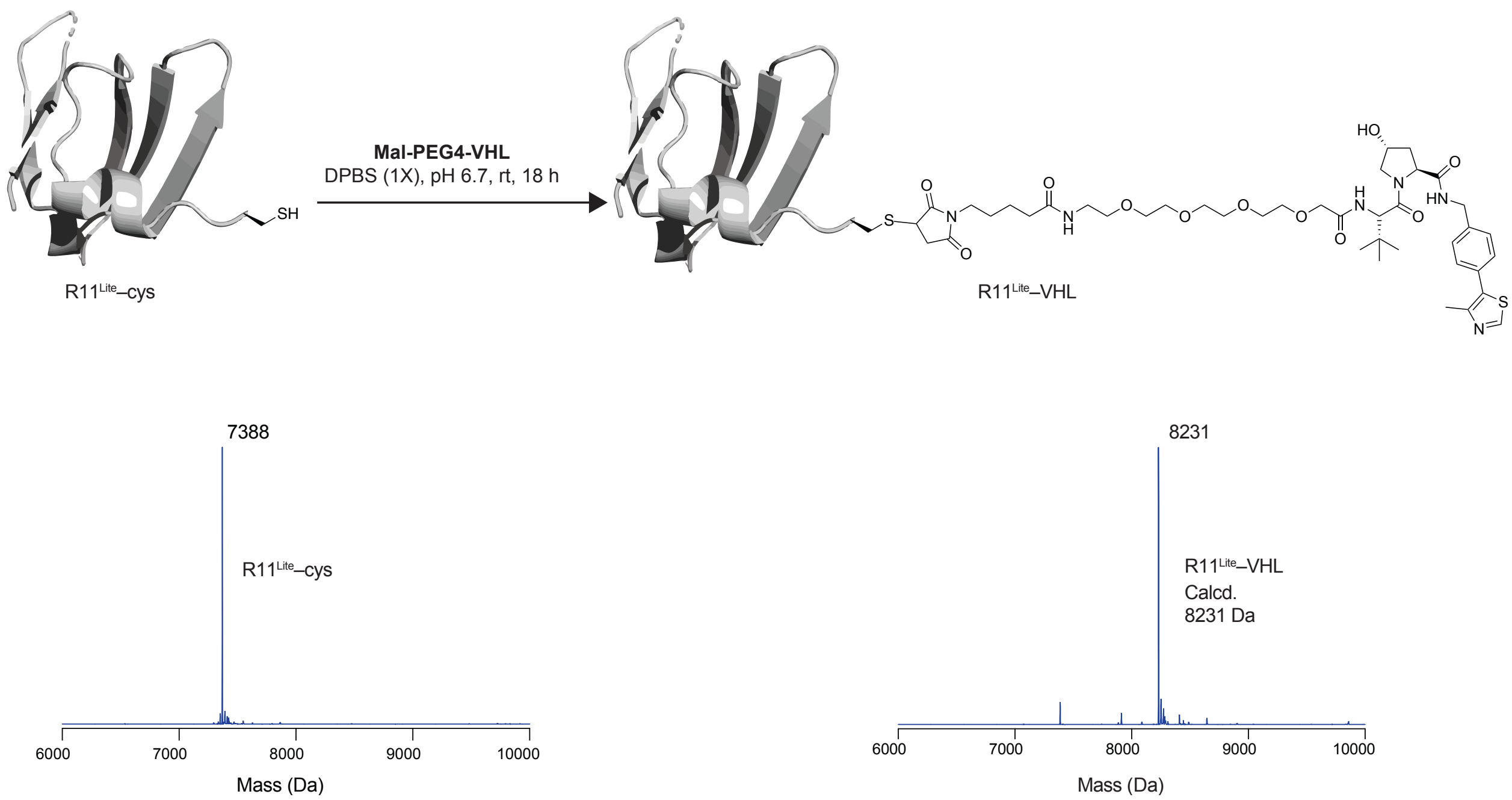

FIGURE S6 Preparation of R11^{Lite}–VHL and deconvoluted ESI Q-TOF mass spectra showing R11^{Lite}-Cys (left) and R11^{Lite}–VHL (right).

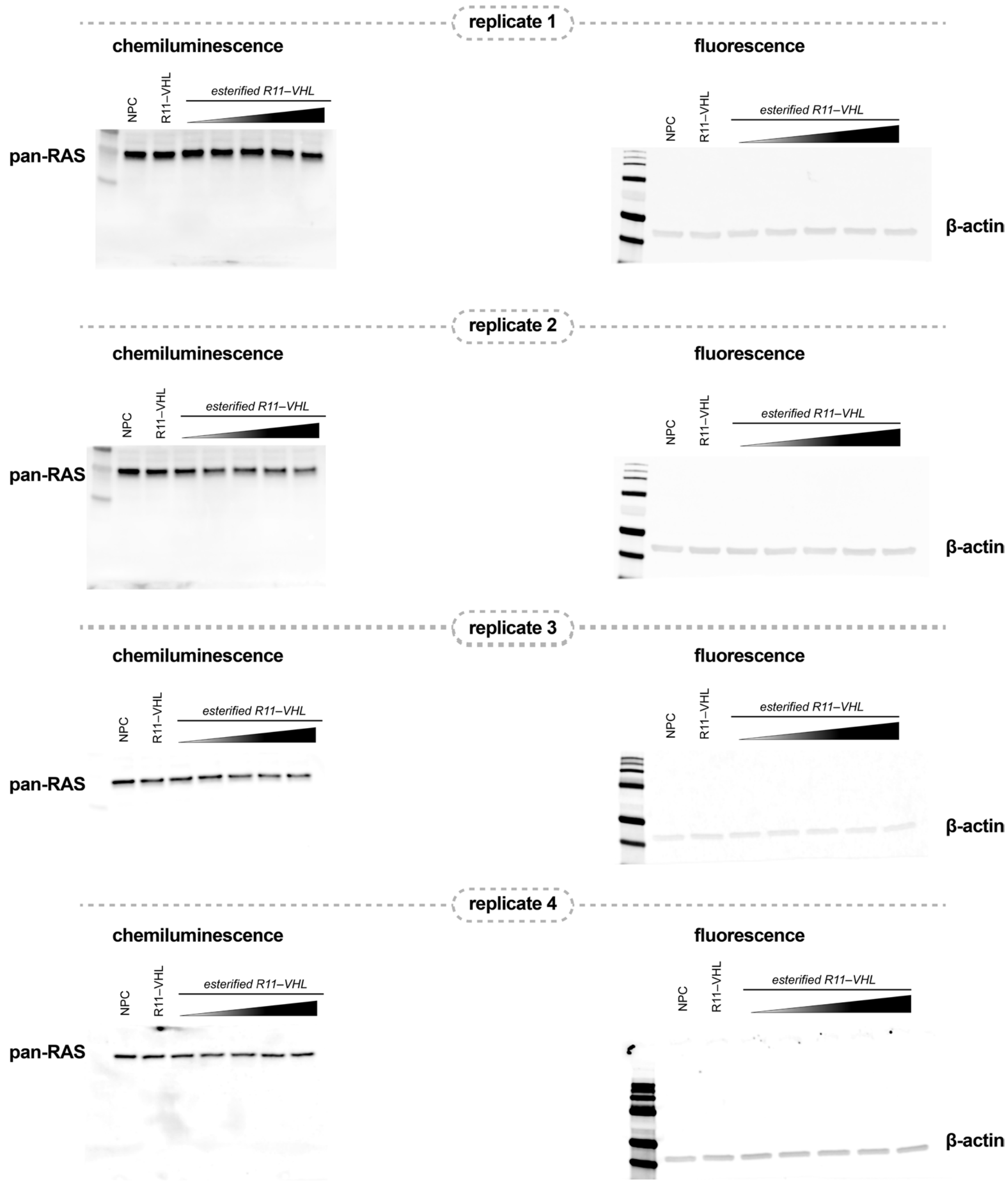

FIGURE S7 Immunoblots of HPAF-II cells treated with esterified R11–VHL. Various concentrations of esterified R11–VHL (0.5, 1.0, 2.5, 5.0, and 10.0 μM) were incubated with cells for 24 h prior to cell harvesting and cell lysis. Cells were treated with unesterified R11–VHL at 10.0 μM as a control. Levels of pan-RAS proteins were probed using a pan-RAS antibody. The loading control was β-actin. These blots were used to generate the data shown in Figure 4A. NPC, no protein control.

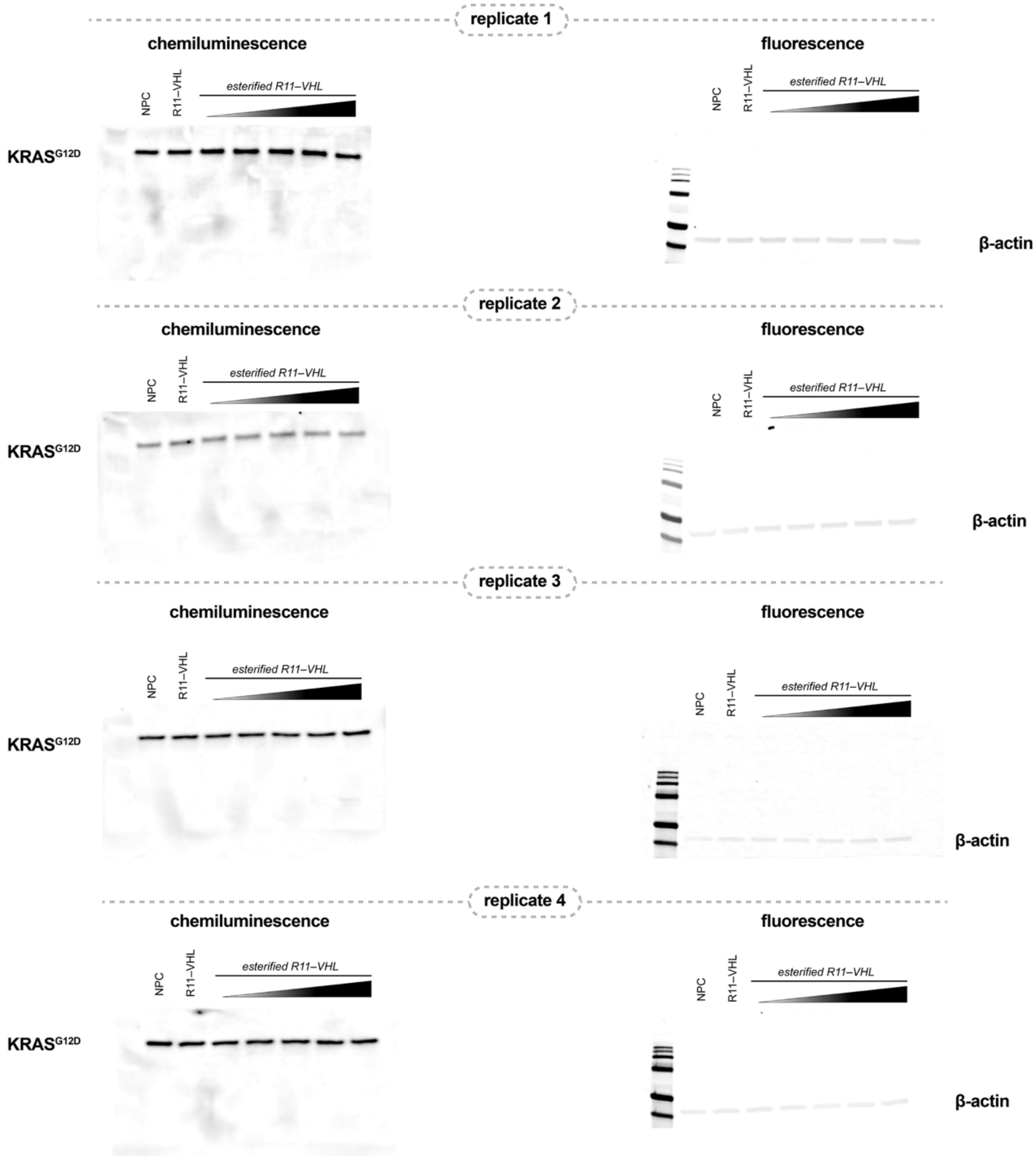

FIGURE S8 Immunoblots of HPAF-II cells treated with esterified R11–VHL. Various concentrations of esterified R11–VHL (0.5, 1.0, 2.5, 5.0, and 10.0 μM) were incubated with cells for 24 h prior to cell harvesting and cell lysis. Cells were treated with unesterified R11–VHL at 10.0 μM as a control. Levels of KRAS^{G12D} were detected using a KRAS^{G12D} antibody. The loading control was β-actin. These blots were used to generate the data shown in Figure 4A. NPC, no protein control.

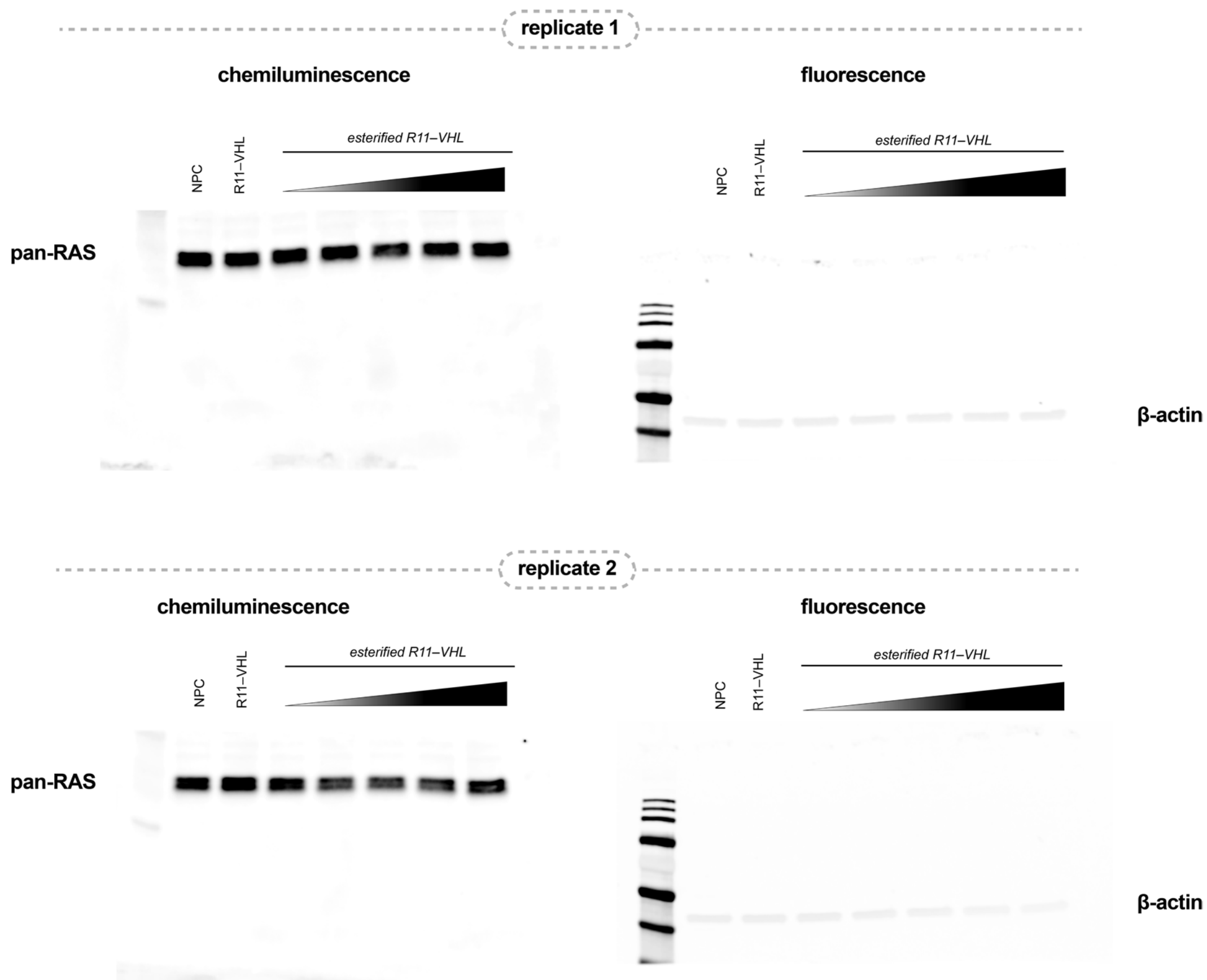

FIGURE S9 Immunoblots of AsPC-1 cells treated with esterified R11–VHL. Various concentrations of esterified R11–VHL (0.5, 1.0, 2.5, 5.0, and 10.0 μM) were incubated with cells for 24 h prior to cell harvesting and cell lysis. Cells were treated with unesterified R11–VHL at 10.0 μM as a control. Levels of pan-RAS proteins were detected using a pan-RAS antibody. The loading control was β-actin. These blots were used to generate the data shown in Figure 4B. NPC, no protein control.

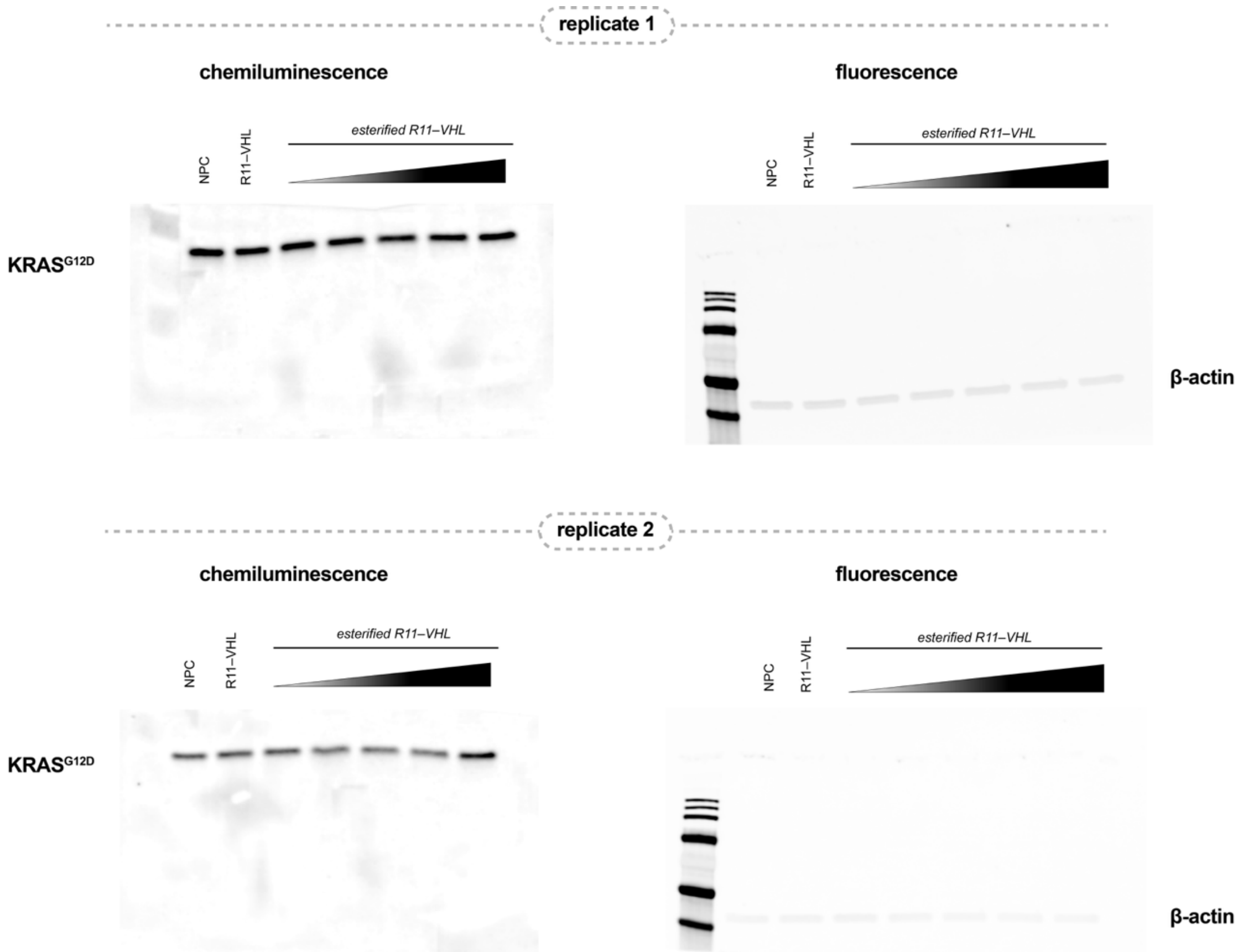

FIGURE S10 Immunoblots of AsPC-1 cells treated with esterified R11–VHL. Various concentrations of esterified R11–VHL (0.5, 1.0, 2.5, 5.0, and 10.0 μM) were incubated with cells for 24 h prior to cell harvesting and cell lysis. Cells were treated with unesterified R11–VHL at 10.0 μM as a control. Levels of $KRAS^{G12D}$ were detected with a $KRAS^{G12D}$ antibody. The loading control was β-actin. These blots were used to generate the data shown in Figure 4B. NPC, no protein control.

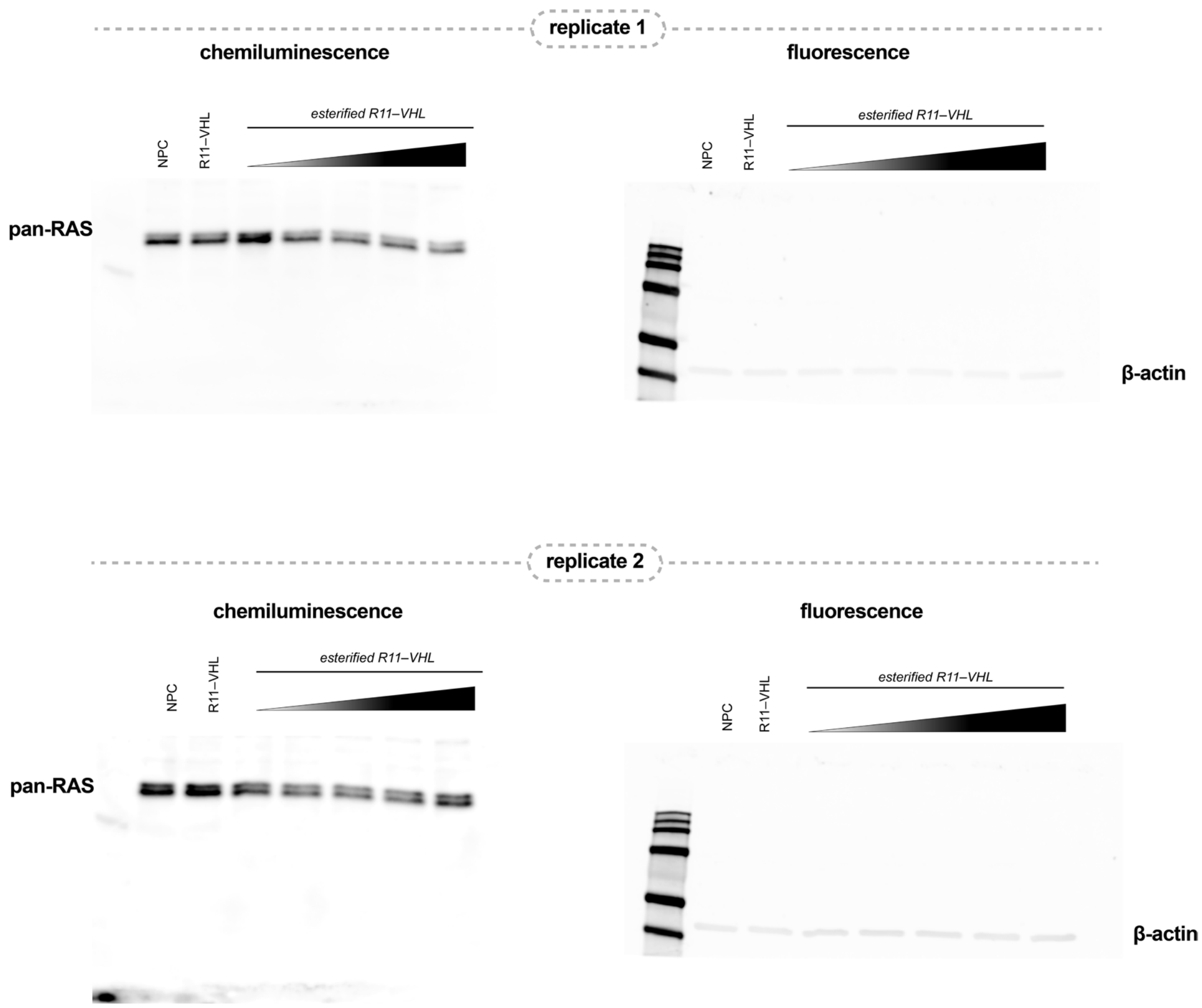

FIGURE S11 Immunoblots of SW48^{G12D} cells treated with esterified R11–VHL. Various concentrations of esterified R11–VHL (0.5, 1.0, 2.5, 5.0, and 10.0 μM) were incubated with cells for 24 h prior to cell harvesting and cell lysis. Cells were treated with unesterified R11–VHL at 10.0 μM as a control. Levels of pan-RAS proteins were detected using a pan-RAS antibody. The loading control was β-actin. These blots were used to generate the data shown in Figure 4C. NPC, no protein control.

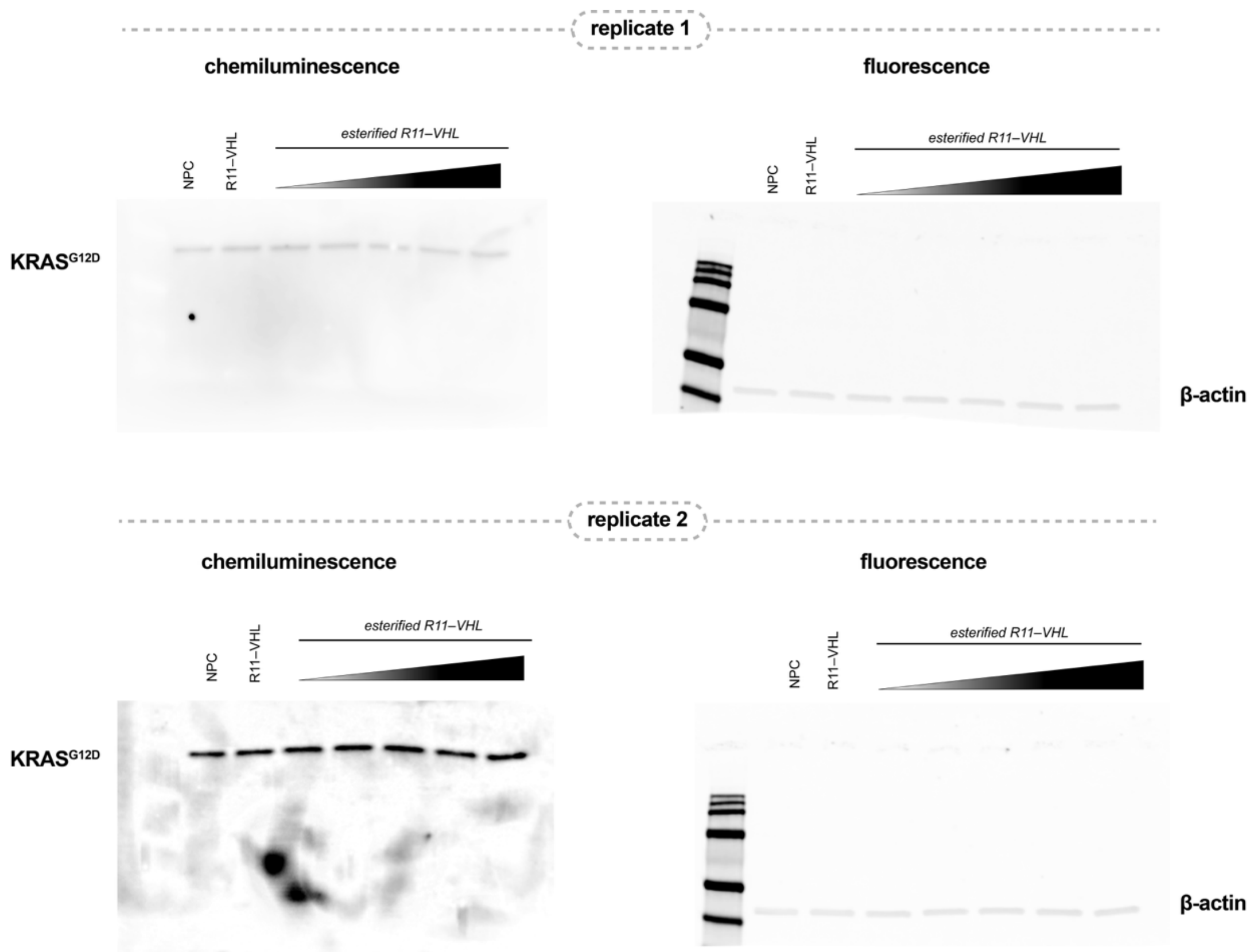

FIGURE S12 Immunoblots of $SW48^{G12D}$ cells treated with esterified R11–VHL. Various concentrations of esterified R11–VHL (0.5, 1.0, 2.5, 5.0, and 10.0 µM) were incubated with cells for 24 h prior to cell harvesting and cell lysis. Cells were treated with unesterified R11–VHL at 10.0 µM as a control. Levels of $KRAS^{G12D}$ were detected with a $KRAS^{G12D}$ antibody. The loading control was β-actin. These blots were used to generate the data shown in Figure 4C. NPC, no protein control.

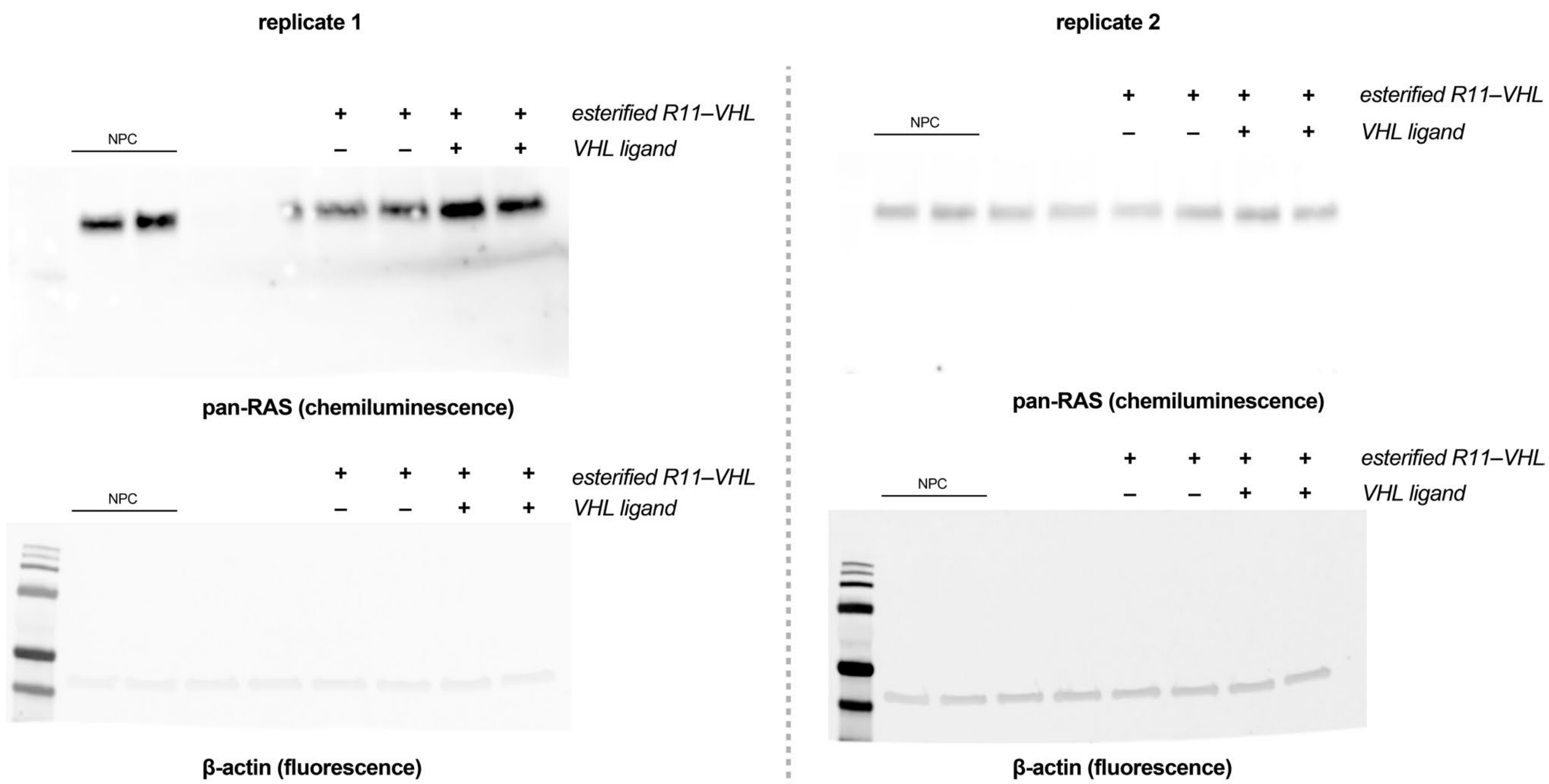

FIGURE S13 Immunoblot of competition study with esterified R11–VHL and VHL ligand in HPAF-II cells. Cells were treated with 10.0 μM R11–VHL in the presence or absence of VHL ligand (200 μM) for 24 h prior to cell harvesting and cell lysis. Levels of pan-RAS proteins were detected using a pan-RAS antibody. The loading control was β-actin. These blots were used to generate the data shown in Figure 5. NPC, no protein control.

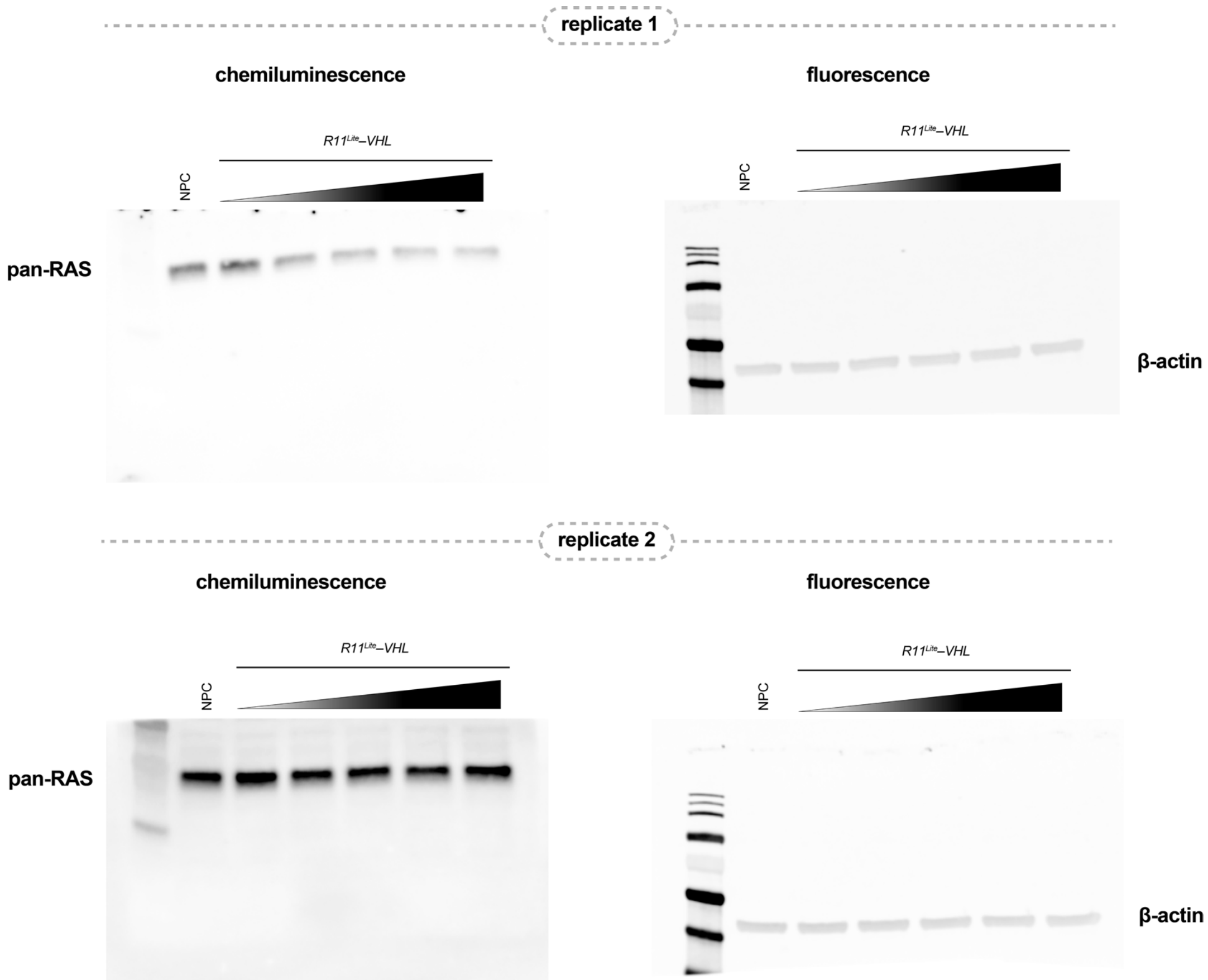

FIGURE S14 Immunoblots of HPAF-II cells treated with R11^{Lite}–VHL. Various concentrations of R11^{Lite}–VHL (0.5, 1.0, 2.5, 5.0, and 10.0 μM) were incubated with cells for 24 h prior to cell harvesting and cell lysis. Levels of pan-RAS proteins were detected using a pan-RAS antibody. The loading control was β-actin. These blots were used to generate the data shown in Figure S15. NPC, no protein control.

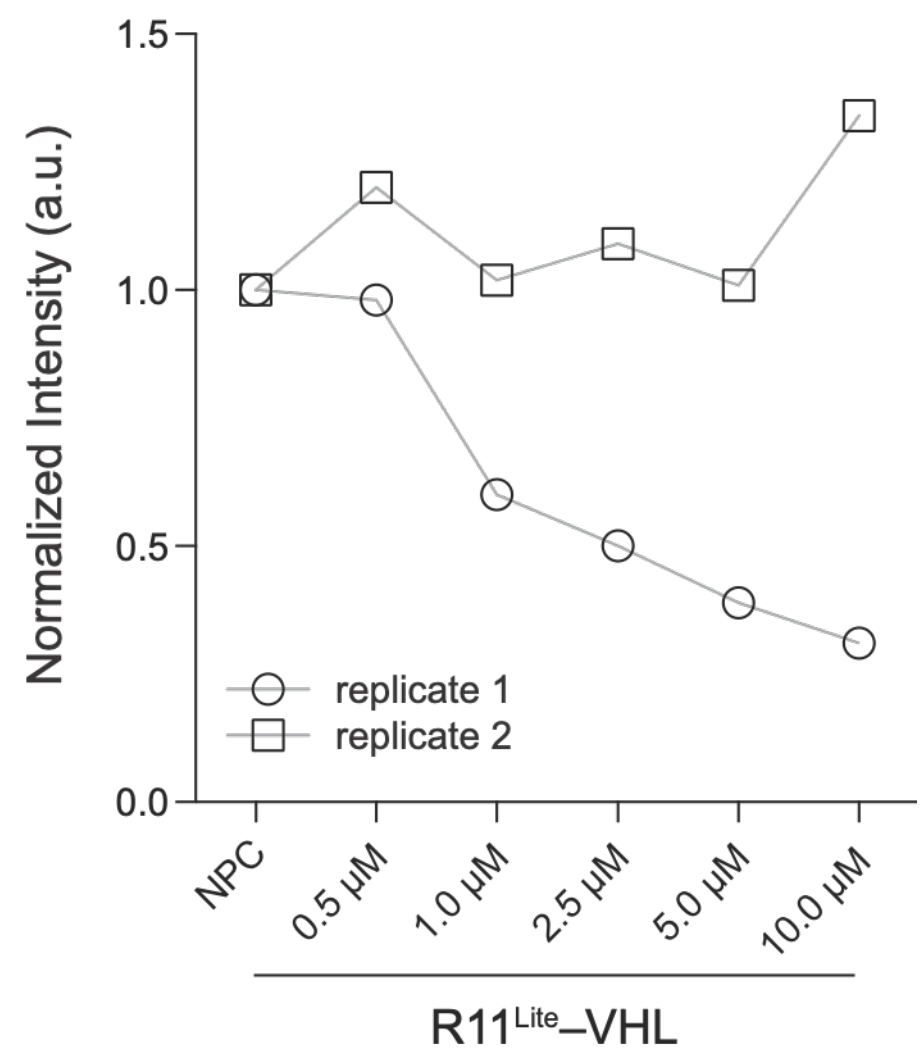

FIGURE S15 Quantification of pan-RAS levels in HPAF-II cells treated with the indicated concentrations of R11^{Lite}–VHL for 24 h, normalized to β-actin and to the no-protein control (NPC).

NMR Spectra

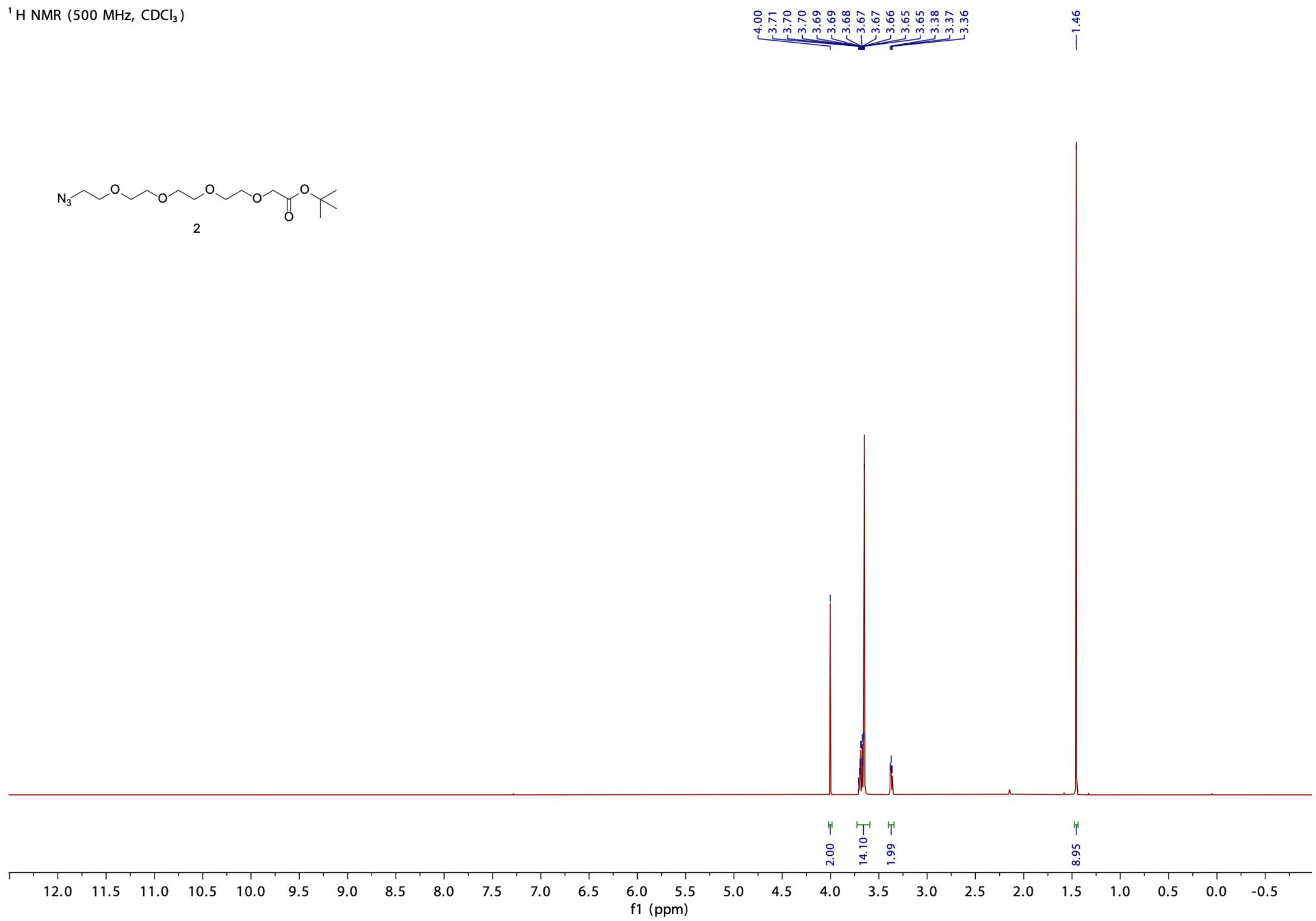
¹H NMR (500 MHz, CDCl₃)
4.00 3.71 3.70 3.70 3.69 3.69 3.68 3.67 3.67 3.66 3.65 3.65 3.38 3.37 3.36
1.46
N3
O
2
2.00
14.10
1.99
8.95
12.0 11.5 11.0 10.5 10.0 9.5 9.0 8.5 8.0 7.5 7.0 6.5 6.0 5.5 5.0 4.5 4.0 3.5 3.0 2.5 2.0 1.5 1.0 0.5 0.0 -0.5
f1 (ppm)

^{13}C NMR (126 MHz, $CDCl_3$)

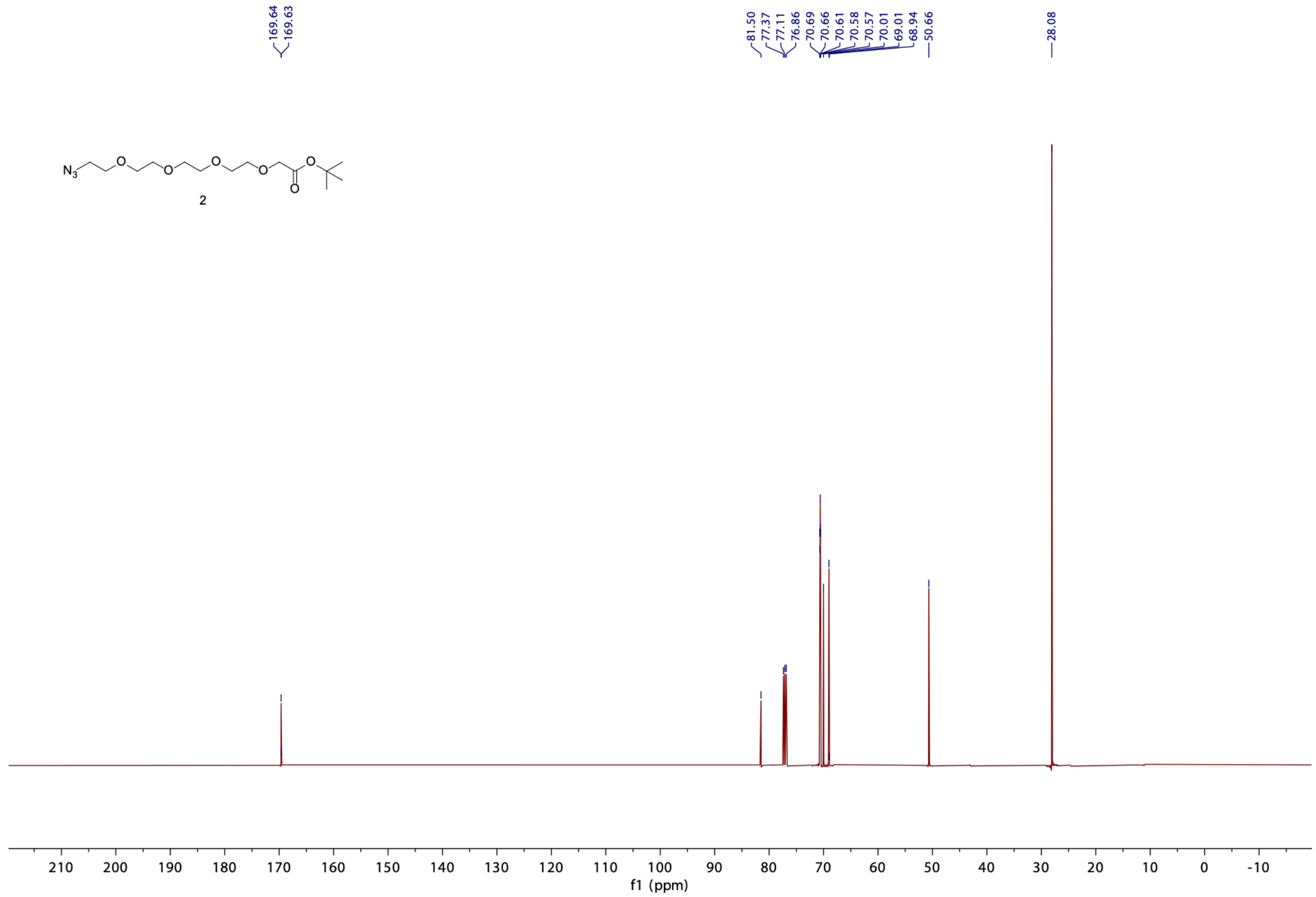

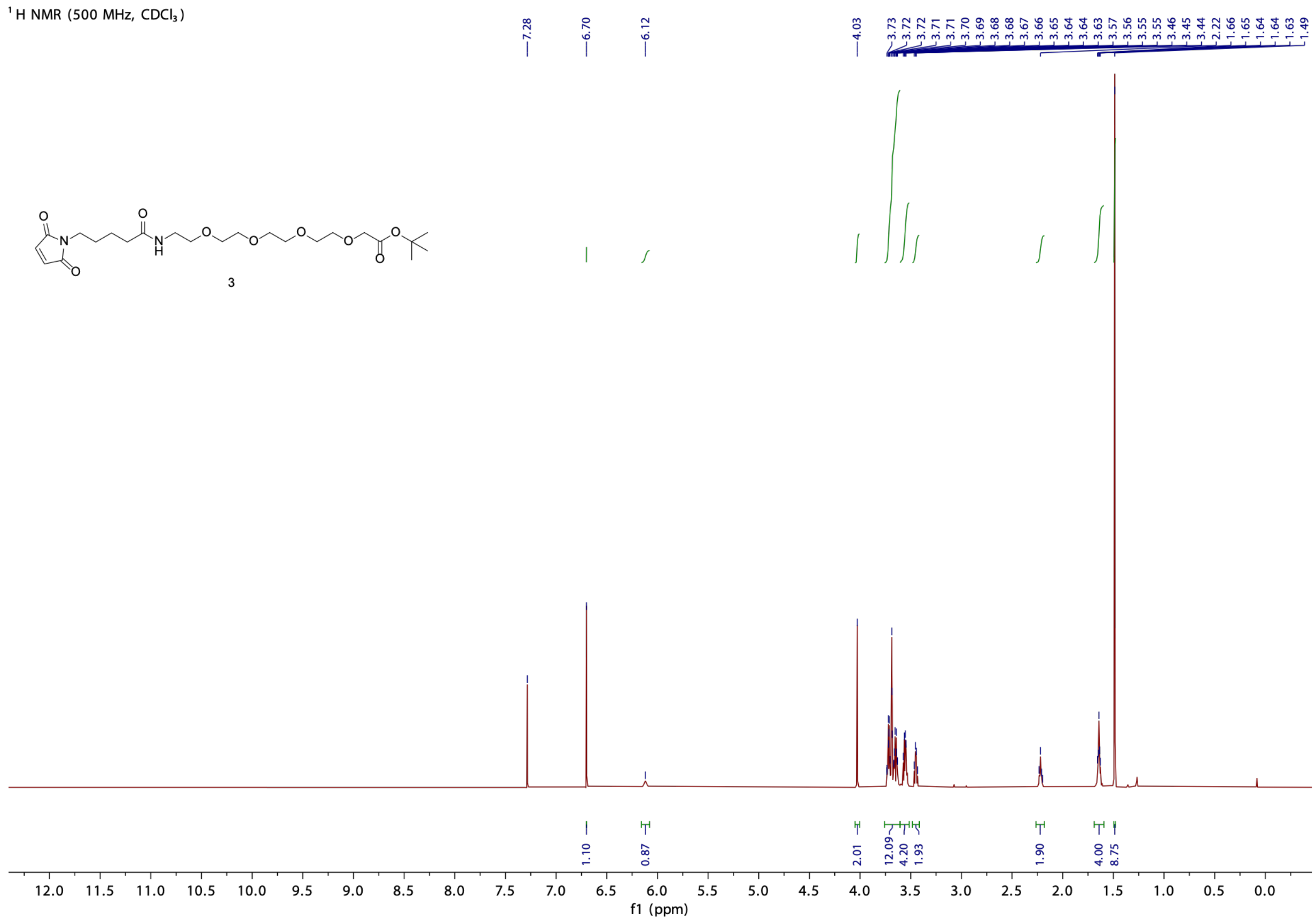

1 H NMR (500 MHz, CDCl3)
3
7.28
6.70
6.12
4.03
3.73 3.72 3.72 3.71 3.71 3.70 3.69 3.68 3.68 3.67 3.66 3.65 3.64 3.64 3.63 3.57 3.56 3.55 3.55 3.46 3.45 3.44 2.22 1.66 1.65 1.64 1.64 1.63 1.49
1.10
0.87
2.01
12.09
4.20
1.93
1.90
4.00
8.75
12.0 11.5 11.0 10.5 10.0 9.5 9.0 8.5 8.0 7.5 7.0 6.5 6.0 5.5 5.0 4.5 4.0 3.5 3.0 2.5 2.0 1.5 1.0 0.5 0.0
f1 (ppm)

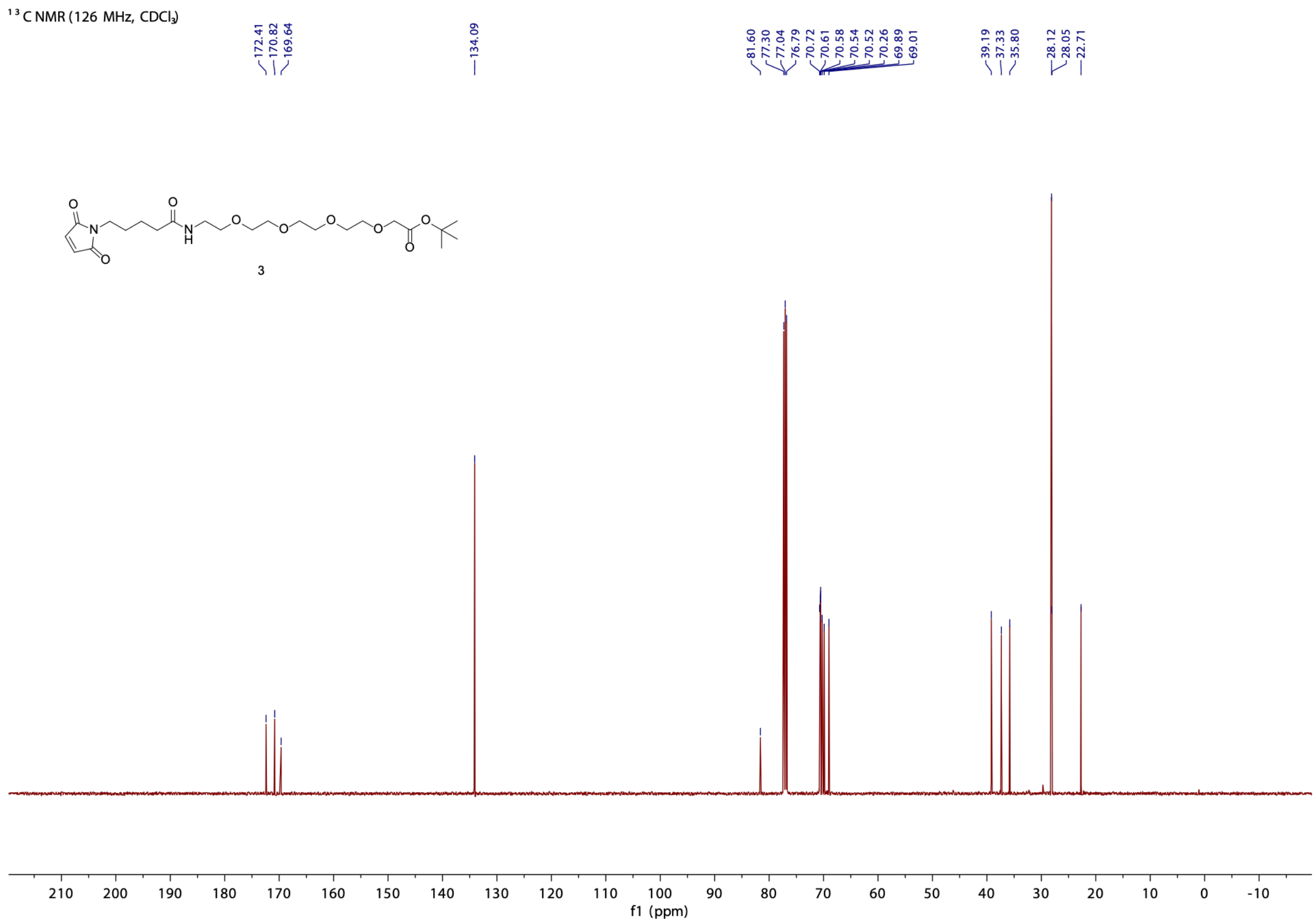

^{13}C NMR (126 MHz, CDCl$_3$)
172.41
170.82
169.64
134.09
81.60
77.30
77.04
76.79
70.72
70.61
70.58
70.54
70.52
70.26
69.89
69.01
39.19
37.33
35.80
28.12
28.05
22.71
3
f1 (ppm)

^{1}H NMR (500 MHz, $CDCl_3$)

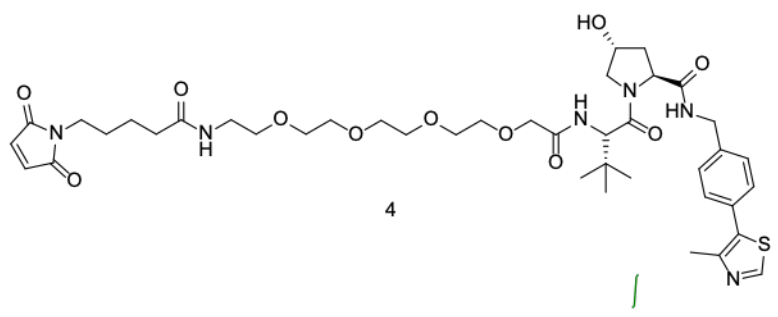

4

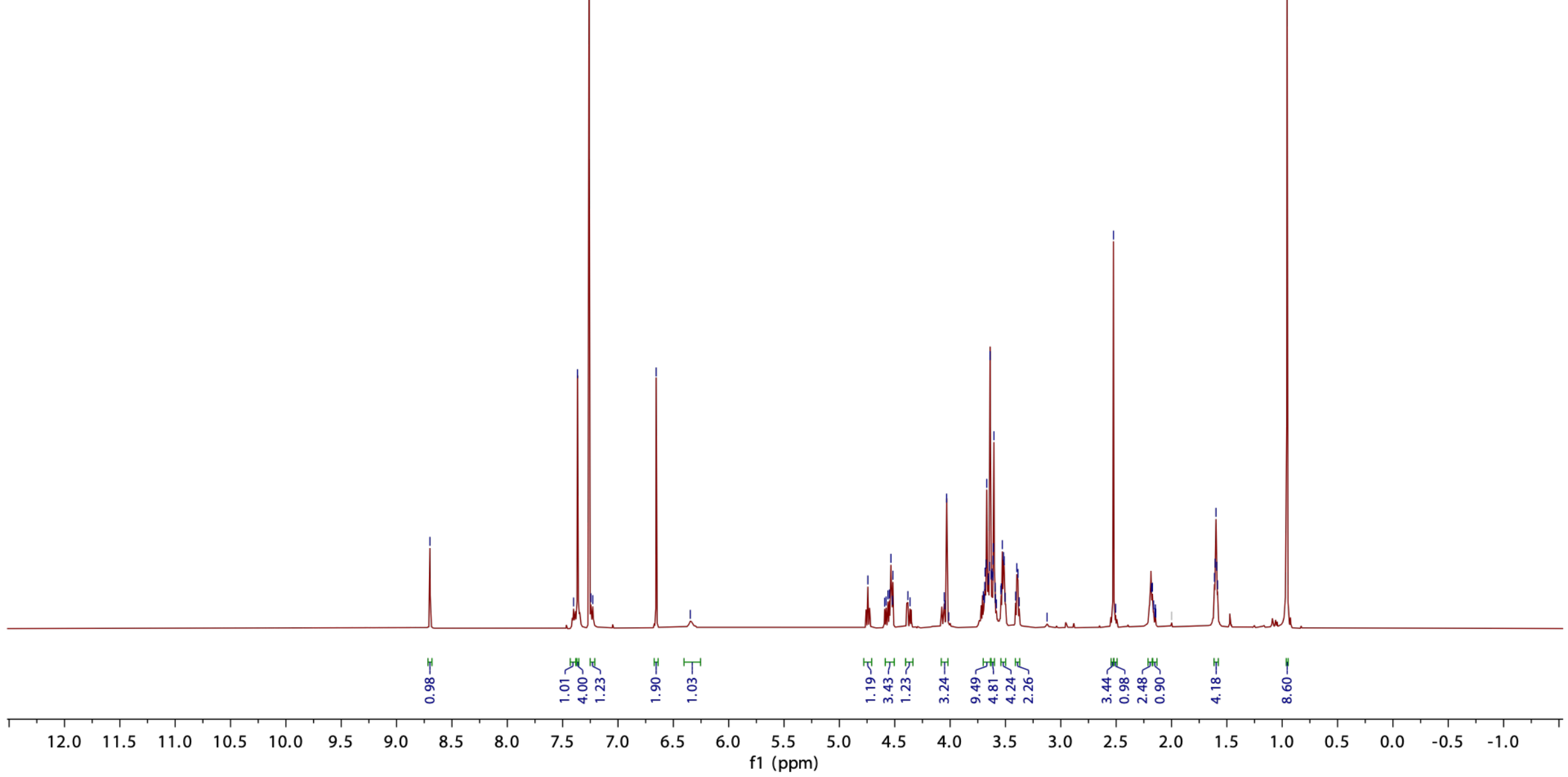

^{13}C NMR (126 MHz, $CDCl_3$)

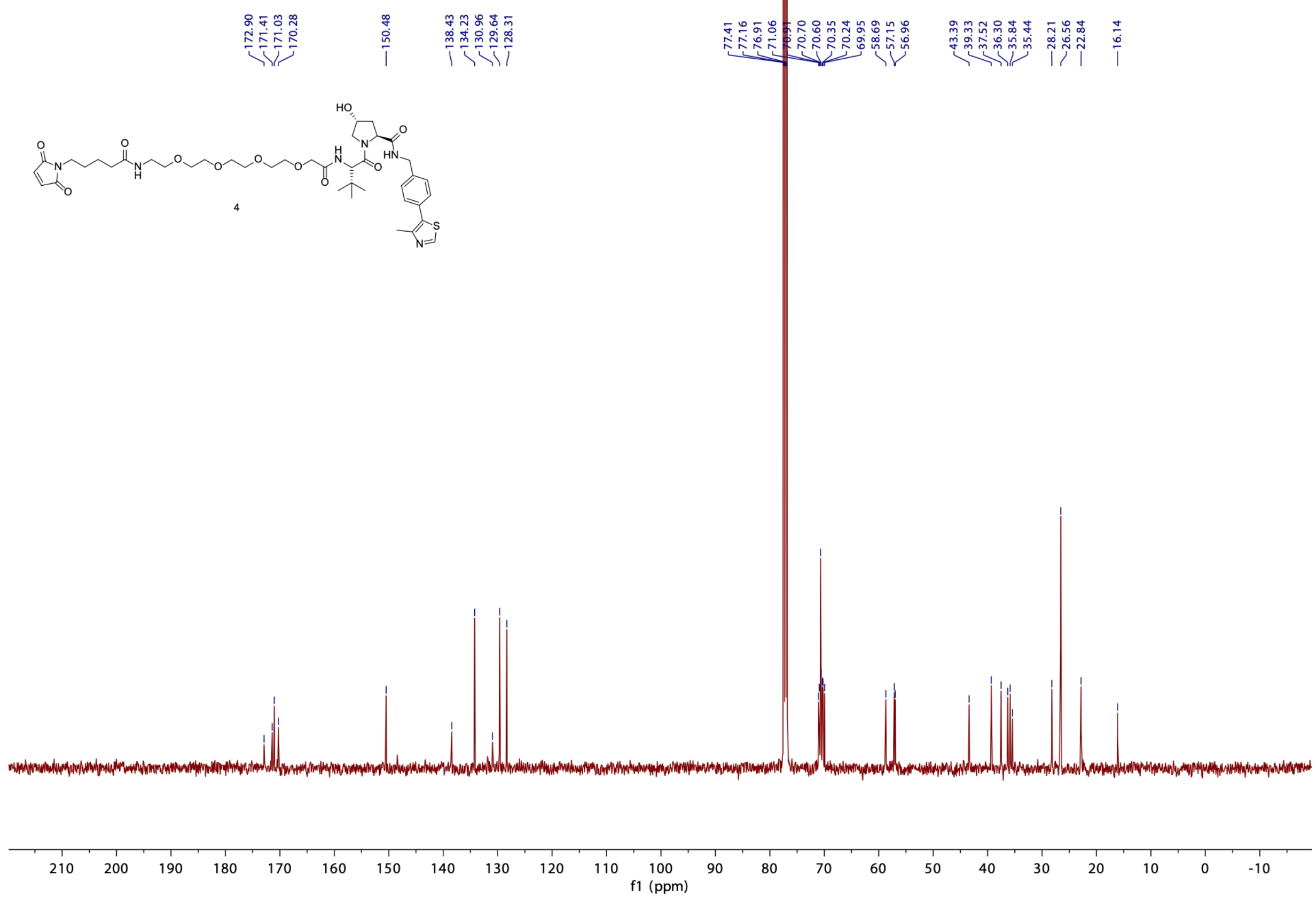

Supplement: Supplementary file 1 [file PRO-35-e70850-s001.pdf]
